# Genomic, transcriptomic, and T cell receptor profiling in stratifying response to first-line chemoradiotherapy or radiotherapy for esophageal squamous cell carcinoma

**DOI:** 10.3389/fonc.2024.1495200

**Published:** 2025-01-06

**Authors:** Xiaqin Zhang, Jianhong Lian, Fukun Chen, Kai Wang, Haoyuan Xue, Sufang Jia, Weili Wang, Zhongkang Li, Hua Liang, Hongwei Li

**Affiliations:** ^1^ Department of Radiotherapy, Shanxi Province Cancer Hospital/Shanxi Hospital Affiliated to Cancer Hospital, Chinese Academy of Medical Sciences/Cancer Hospital Affiliated to Shanxi Medical University, Taiyuan, Shanxi, China; ^2^ Department of Thoracic Surgery, Shanxi Province Cancer Hospital/Shanxi Hospital Affiliated to Cancer Hospital, Chinese Academy of Medical Sciences/Cancer Hospital Affiliated to Shanxi Medical University, Taiyuan, Shanxi, China; ^3^ Geneplus-Beijing, Beijing, China; ^4^ Shanxi Medical University, Taiyuan, Shanxi, China; ^5^ Ludwig Center for Metastasis Research, Department of Radiation and Cellular Oncology, University of Chicago, Chicago, IL, United States

**Keywords:** esophageal squamous cell carcinoma, transcriptome, T-cell receptor analysis, radiotherapy, multi-omics analysis esophageal squamous cell carcinoma, multi-omics analysis

## Abstract

**Introduction:**

Esophageal squamous cell carcinoma (ESCC) accounts for 80% of esophageal cancer (EC) worldwide. The molecular characteristics of locally advanced ESCC have been extensively studied.

**Methods:**

In this study, we investigate the genomic and transcriptomic characteristics and try to provide the basic T-cell receptors (TCRs) dynamics and its genomic and transcriptome association during the radiochemotherapy of ESCC using multi-omics analysis.

**Results:**

A total of 23 patients with pathologic diagnoses of locally advanced ESCC were enrolled. The median tumor mutational burden (TMB) of the 23 ESCC patients were 3.47 mutations/ Mb (mega-base). The TP53, RTK/RAS, and NOTCH pathways were concurrently prevalent in ESCC. Besides, some less prevalent pathways, including WNT and HIPPO pathways also exhibited superior frequencies in ESCC. Meantime, we found the immune-hot tumor had higher immune infiltration scores. The median TMB in the progression-free survival (PFS) low group was significantly higher than that in the PFS-high group. The chromosomal copy number variation (CNV) burden of the neutrophil-to-lymphocyte ratio (NLR)-high group appeared to be higher than that of the NLR-low group, and the StromalScore in the NLR-low group was significantly higher. Clonality score was significantly increased from pre-treat to post-treat and from on-treat to post-treat. Shannon index was significantly decreased from pre-treat to post-treat and from on-treat to posttreat. Richness was significantly decreased from pre-treat to post-treat.

**Discussion:**

Multiomics analysis provided the basic TCRs dynamics and their genomic and transcriptome association during the radio-chemotherapy of 23 locally advanced ESCC in China, and provided a valuable insights into the heterogeneity and the tumor microenvironment and treatment responses. Meantimes, the identification of biomarkers and the exploration of their association with treatment outcomes could have important implications for clinical practice.

## 1 Introduction

Esophageal cancer (EC) is the tenth most common malignancy and the sixth most common cause of cancer death in the world (1). In China, it accounts for the fifth in morbidity and fourth in mortality across all cancers (2). Esophageal squamous cell carcinoma (ESCC), the most common histology, accounts for 80% of EC worldwide and is more prevalent in Asia, Africa, and South America, with more than half of ESCC cases occurring in China (3–6). For those ESCC patients with locally advanced disease who are inoperable at the time of diagnosis, radiotherapy, definitive chemo-radiation therapy (CRT) and the sequential use of radiotherapy and chemotherapy is first-line therapies (7–9). Despite the remarkable efficacy of radiotherapy and chemotherapy (7–9), there is an urgent need for biological and molecular characterization of the tumor microenvironment that may affect the efficacy of chemoradiotherapy in locally advanced ESCC patients.

To identify the molecular aberrations that drive ESCC tumorigenesis and progression, extensive genomic, epigenomic, and transcriptomic research has been conducted by The Cancer Genome Atlas (TCGA) and other organizations (10). Based on the multi-omics profiling, potential therapeutic targets and diagnostic markers have been identified, and additional resources could be provided for future investigations on ESCC. Recently, some therapeutic targets, predictive and prognostic biomarkers and molecular classification has been identified in ESCC (11–13). However, the relationship between genomic characteristics and radiotherapy in patients with ESCC has not been explored in depth. In addition, antigen peptides are recognized by specific T-cell receptors (TCRs) in T cells, which are expressed on their surface. The specificity of the TCR is determined primarily by complementarity determining region 3 (CDR3) which is highly variable (14). Studies have shown that CDR3 diversity has a significant role in cancer diagnosis, therapy, and prognosis, since it reflects the diversity of cellular immunity (14–16). Analyzing TCR evolution dynamics before and after treatment in patients not only enhances our understanding of the mechanisms of effective or ineffective anticancer treatment but also provides improved direction for anticancer treatment.

In this study, we aimed to reveal the genomic, transcriptomic, and TCR dynamics before and after radiotherapy of locally advanced ESCC in depth. We collected tumor tissue samples from the enrolled population for whole-exome sequencing (WES) and RNA sequencing to identify mutations, copy number variations, hallmark oncogenic pathways, and immune microenvironment characteristics of ESCC. Subsequently, we performed TCR sequencing on peripheral blood samples before, during, and after radiotherapy, and it was identified that patients with increased TCR diversity during radiotherapy had better progression-free survival (PFS). This study provides prognostics of therapeutic effectiveness markers for patients receiving radiotherapy. Finally, this study explores the associations between different omics, providing new insights into future treatment responses for esophageal cancer.

## 2 Materials and methods

### 2.1 Patients and samples

A total of 23 patients with a definite diagnosis of ESCC were enrolled from March 2021 to December 2021 in this study. Clinicopathological information, including demographics, pathologic diagnoses, imaging examinations, and treatment history were collected from each patient. A total of 8 patients received radiotherapy alone and 15 patients received a combination of radio-chemotherapy, as detailed in **Table 1**, the chemotherapy regimen was based on clinical guidelines, mainly with nedaplatin and Tegafur Gimer. Tumor tissue samples were collected from all participants who received radiotherapy to perform the whole exome (WES) and transcriptome (WTS) sequencing and matched peripheral blood samples which included three-time points, pre-treatment, on-treatment, and post-treatment were collected to perform the TCR-βsequencing. All procedures were conducted by the Declaration of Helsinki. This study was approved by the Ethics Committee of Shanxi Provincial Cancer Hospital (Taiyuan, China) (Approval No: KY2022011) and written informed consent was obtained from all participants.

**Table 1:** Clinicopathologic characteristics of 23 patients with locally advanced ESCC.

Characteristics	Patients (n=23)
Age at diagnosis-years	
Median (range)	69 (53-81)
Gender-No. (%)	
Male	11 (47.8%)
Female	12 (52.2%)
Disease stage- No. (%)	
II	7 (30.4%)
III	9 (39.2%)
IV	7 (30.4%)
Lymph node metastasis positive - No. (%)	
Yes	19 (82.6%)
No	4 (17.4%)
Personal history -No. (%)	
Smoking history	10 (43.5%)
Drinking history	6 (26.1%)
Family history	0 (0%)
Therapy-No. (%)	
Radiotherapy	8 (34.8%)
Radio-chemotherapy	15 (65.2%)
NLR	
Median (range)	1.79 (0.93-8.47)
PFS- months	
Median (range)	14 (1-27)

NLR, neutrophil-to-lymphocyte ratio; PFS, progression free survival.

### 2.2 WES, WTS and T-cell receptor–β library construction and sequencing

DNA- and RNA-based NGS were performed in Geneplus-Beijing (Beijing, China) using WES and WTS as published in our previous study (17, 18). Genomic DNA (gDNA) from WBCs and tumor tissues were processed into indexed libraries using a DNeasy Blood & Tissue Kit (Qiagen, Hilden, Germany). RNA was extracted from the tumor samples using an RNeasy FFPE Kit (Qiagen, Hilden, Germany). Sequencing libraries of genomic DNA and mRNA were prepared using the KAPA DNA Library Preparation Kit (Kapa Biosystems, MA, USA) and NEB Next Ultra™ RNA Library Prep Kit (Illumina, Inc., CA, USA), respectively. The DNA and RNA sequencing were performed and all experimental procedures followed the manufacturer’s instructions. The DNA and RNA indexed libraries were sequenced using a 100-bp paired-end configuration on a DNBSEQ-T7RS sequencer (MGI Tech, Shenzhen, China) or Gene+Seq-2000 sequencing system (GenePlus, Suzhou, China), producing 30G(150X), 12G (150X), 12G sequencing data for tumor tissues (WES), WBLs (WES), and tumor tissues (RNA), respectively. TCR sequencing and data analysis were performed in Geneplus-Beijing (Beijing, China) as previously described (14, 16). Multiplex PCR amplification of CDR3 of the TCR-β chain (TRB) was conducted including PCR1 and PCR2, inclusively and semi-quantitatively. Libraries were sequenced using the PE150 strategy on the Gene+Seq-2000 sequencing system (GenePlus, Suzhou, China), producing 2G/sample. Based on the ImMunoGeneTics (IMGT) V, D, and J gene references, the CDR3 sequence is characterized as the amino acids situated between the second cysteine in the V region and the conserved phenylalanine in the J region. The MiXCR software package is employed to identify and allocate CDR3 sequences (19).

### 2.3 Bioinformatics analysis

After the removal of terminal adaptor sequences and low-quality reads (>50% N rate, >50% bases with Q<5) by FASTP (v0.12.6) (20), the remaining reads were aligned to the reference human genome (hg19) and aligned using BWA (version 0.7.10) (21) and HISAT (22) for DNA and RNA sequencing, respectively. Duplicated reads were removed using the MarkDuplicates tool in Picard (version 4.0; Broad Institute). Genomic single nucleotide variants (SNVs), small insertions and deletions (InDels), copy number variants (CNVs), and structural variants (SVs) were called with default parameters by MuTect (version 1.1.4) (23)/NChot, GATK (v3.6-0-g89b7209; Broad Institute) and CONTRA (version 2.0.8) (24), respectively. Transcript assembly was performed using StringTie (version 1.2.3) (25). Chromosomal CNV burden represented the total level of amplifications or deletions at the chromosome level. Significantly recurrent regions with amplification or deletion were detected using Gistic 2.0 with a noise threshold of 0.3, a broad length cutoff of 0.5 chromosome arms, a confidence level of 95%, and a copy-ratio cap of 1.5 (26). The mutational landscape was portrayed using the R package ‘maftools’ (version 2.14.0). The CDR3 sequences were identified and assigned using the MiXCR software package (version 3.0.3) (19). The relative abundance or distribution of each clonotype. Shannon’s entropy was calculated on the clonal abundance of all productive TCR sequences. Clonality score is defined as 1- (Shannon index)/ln(# of productive unique sequences) (16).

Non-synonymous SNVs and Indels with a mutant allele frequency greater than 5% per megabase in the coding region were included in the calculation of Tumor mutation burden (TMB). R package ‘yapsa’ (version 1.24.0) was performed to infer the composition of known Catalogue of Somatic Mutations in Cancer (COSMIC) mutational signatures in EC using the COSMIC mutational signatures version 2 (27). Normalization, estimation of dispersion, and statistical testing of differential expression were performed using the DESeq function in DESeq2 (version 1.38.3) with default parameters. Genes with an adjusted p-value (q-value) < 0.05 and an absolute log2 fold-change (log2FC) > 1 were considered as significantly differentially expressed. R package ‘clusterProfiler’ (version 4.7.1.3) was used to perform gene set enrichment analysis (GSEA) enrichment analysis (28) using Kyoto Encyclopedia of Genes and Genomes (KEGG) pathway database (29) and the Gene Ontology Biological Process (GO BP) database (30). Cold and hot tumors were distinguished based on the immune infiltration scores of samples. The approach involved evaluating immune cell scores using the Gaussian calculation of the ssGSEA (28) method from the standardized TPM matrix using GSVA (31). Immune infiltrating cells were activated, suppressed, and classified into other cell types based on their cellular characteristics. The TME deconvolution method from the R package “IOBR” was utilized to assess the immune microenvironment, and the function “iobr_cor_plot” was employed to compute the expression of features or genes and generate plots. The “iobr_cor_plot” function dynamically generates statistical results by processing the calculated score values through the scale function (32).

### 2.4 Statistical analysis

Statistical analysis and visualization were performed using the software R 4.3.2 (R Foundation for Statistical Computing, Vienna, Austria). Sample clustering to distinguish immune-cold and immune-hot tumors was achieved through the Euclidean method within the ConsensusClusterPlus function in software R. For the comparison of TCR dynamics, immune microenvironment, genomic characteristics, and PFS (progression-free survival), as well as the NLR (neutrophil-to-lymphocyte ratio) between groups, a t-test will be performed for variables following a normal distribution, while the Wilcoxon rank-sum test will be applied for non-normally distributed variables.Correlation analysis of different indicators was performed using the Pearson correlation method. A two-tailed P < 0.05 was considered statistically significant.

## 3 Results

### 3.1 Clinicopathologic characteristics

In this study, a total of 23 patients with pathologic diagnoses of locally advanced ESCC were enrolled, and their clinicopathologic features and demographics were summarized in **Table 1**. The median age at diagnosis was 69 years, ranging from 53 to 81 years, with 47.8% (11/23) males. Among them, 43.5% (10/23) and 26.1% (6/23) patients had a smoking and drinking history respectively, and none of the patients had a family history. Of all, 30.4% (7/23) patients had stage II disease, 39.2% (9/23) had stage III and 30.4% (7/23) with stage IV. And 82.6% (19/23) of patients were identified as positive for lymph node metastasis. The lesions were located in the cervical segment in 3 cases, the thoracic segment in 19 cases, and both the stiff segment and thoracic segment in 1 case. The median neutrophil-to-lymphocyte ratio (NLR) was 1.79, ranging from 0.93 to 8.47. Radiotherapy and radio-chemotherapy were conducted in 34.8% (8/23) and 65.2% (15/23) of patients, respectively. Based on a rigorous evaluation of radiological evidence (MRI and radiography) by two independent radiologists, 19 patients achieved complete response (CR) or partial response (PR), and 4 patients were diagnosed with stable disease (SD). The median progression-free survival (PFS) was 14 months, ranging from 1 to 27 months.

### 3.2 Genomic landscape of esophageal squamous cell carcinoma

**Figure 1:**
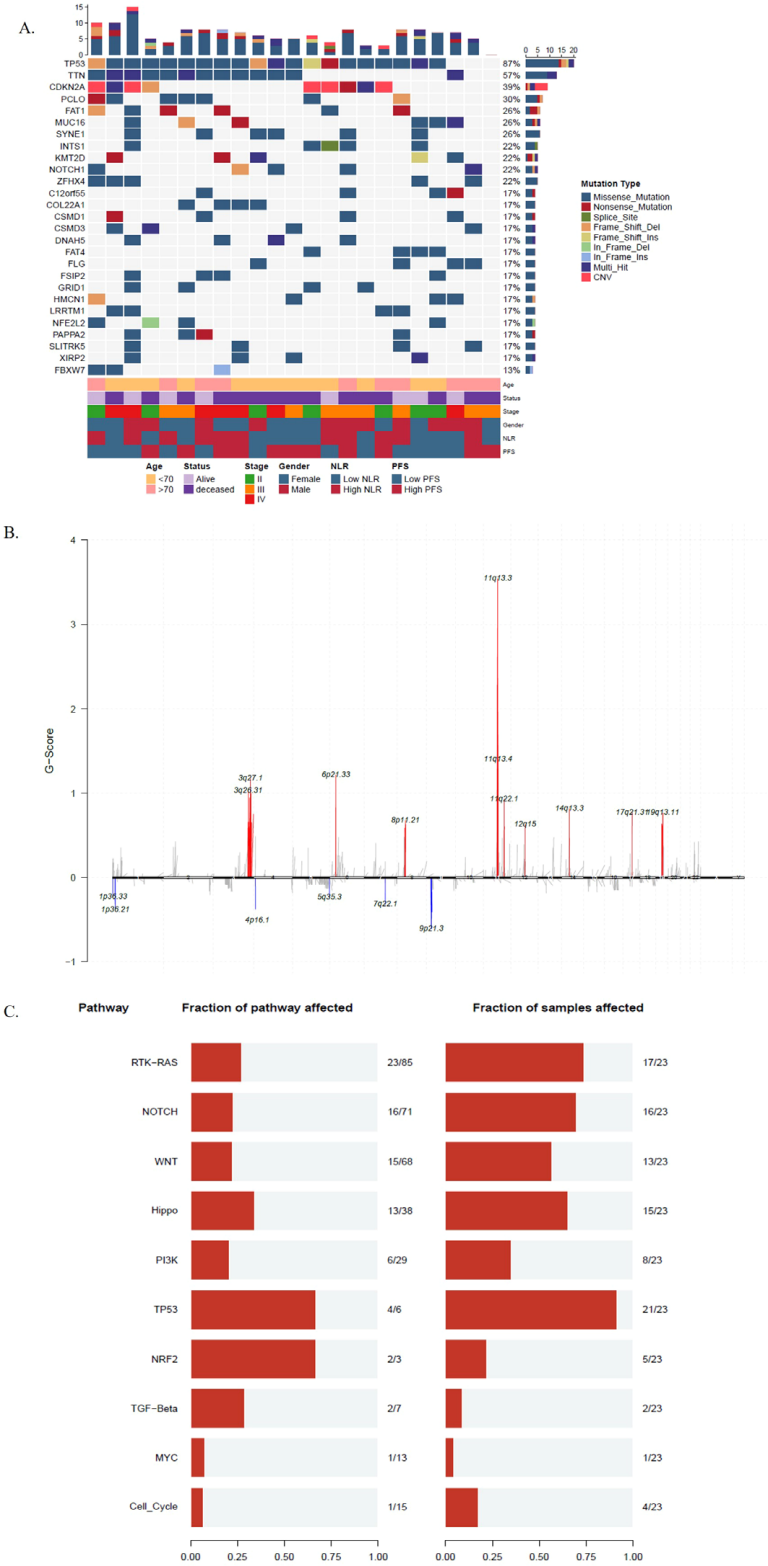
The genomic characteristics of esophageal squamous cell carcinoma (ESCC). **(A)** Clinicopathologic information, mutations and copy number variations landscape of 23 patients with ESCC. The numbers above represent the cumulative counts of different mutation types, while those on the right represent the mutation proportions of different mutation types; **(B)** The y-axis represents the amplitude of copy number variations, with the numerical values indicating the frequency of copy number alterations. The upper portion (in red) indicates copy number amplification, while the lower portion (in blue) indicates copy number deletion; **(C)** Mutant genes of 23 patients with ESCC to 10 hallmark oncogenic pathways. The numbers on the left panel displays the fraction of pathways affected; the right panel shows the fraction of samples affected.

Using WES data, a total of 3517 somatic mutations were detected in 23 patients with ESCC, including 3520 SNVs and 197 Indels. The median number of mutations was 158 (range 22 - 438). *TP53*, *TTN*, *PCLO*, *FAT1*, *MUC16* and *SYNE1* were the top 6 commonly SNVs mutated genes, and mutated in 87%, 57%, 30%, 26%, 26%, and 26% of ESCC, respectively (**Figure 1A**). We detected 143 gene amplifications in these samples, including 89 gene gain alterations and 54 gene loss alterations. The most frequent gene amplifications were of *PLD1*, *TMEM212*, *FNDC3B*, *GHSR*, *TNFSF10*, *NCEH1*, *ECT2*, *SPATA16* and *NLGN1*, and the amplification of these genes were all located on chromosome 3q26.31 (Data not shown).

A total of 11 significantly amplified and 6 deleted regions were identified by Gistic 2.0 (26) with the q value < 0.1 (**Figure 1B**). We also examined the composition of six possible base-pair substitutions by the Catalogue of Somatic Mutations in Cancer (COSMIC) mutational signature analysis, we found that nearly 50% of mutations are C>T (**Supplementary Figure S1A**), and 75% (41% + 34%) of mutational signatures are attributed to either signature 1 (spontaneous deamination of 5−methylcytosine) or signature 2 (APOBEC Cytidine Deaminase), both of which are associated with C>T mutations (**Supplementary Figure S1B**).

The median TMB of the 23 ESCC patients was 3.47 mutations/Mb (range 1-52.86 mutations/Mb). We compared the TMB levels of the 23 ESCC patients in this study with TMB from other cancers derived from TCGA and found that ESCC had a relatively high level of TMB (**Supplementary Figure S2**). Subsequently, we allocated mutant genes to 10 hallmark oncogenic pathways. The TP53, RTK/RAS and NOTCH pathways were consistently prevalent in ESCC (33, 34). Besides, some less prevalent pathways, including WNT and HIPPO pathways also exhibited superior frequencies in ESCC (35). Among distinct pathways, multiple RTK/RAS alterations and WNT alterations tended to be concurrent in one patient (**Figure 1C**).

### 3.3 Immune microenvironment characteristics of esophageal squamous cell carcinoma

**Figure 2:**
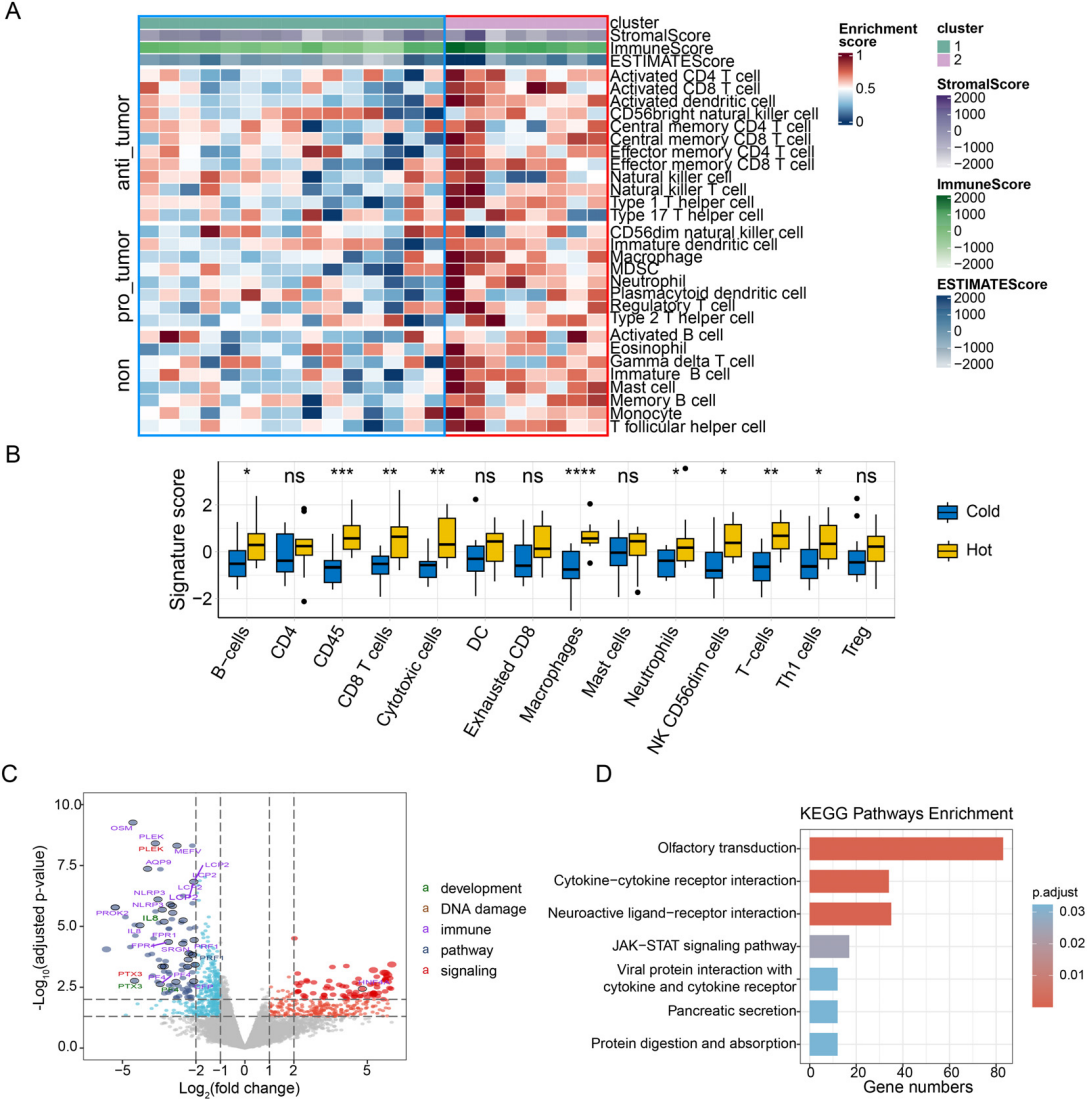
The association of the immune infiltration derived from RNA sequencing with ESCC. **(A)** Heatmap of normalized enrichment scores for infiltration of 28 immune cells, used to classify the 23 ESCC patients into two clusters, immune-hot group (right panel, cluster 2) and immune-cold group (left panel, cluster 1); **(B)** The differences of the composition of immune cells between immune-hot group and immune-cold group. The signature score represents the gene characteristic score of the TME in the samples. **(C)** Volcano plot of differentially expressed genes comparing immune-cold group and immune-hot group. Red dots and blue dots indicate significantly upregulated and downregulated genes in ESCCs, respectively. Categories based on hallmark biological processes define pathways as “development,” “DNA damage,” “immune,” “pathways,” and “signaling”. The size of the points indicates the adjusted p-value. Larger points signify a more significant adjusted p-value; **(D)** KEGG enrichment pathway of the immune-cold group. The “gene number” indicates the number of genes present in each pathway; ESCC, esophageal squamous cell carcinoma; KEGG, kyoto encyclopedia of genes and genomes; ns, p≥0.05; *, p<0.05; **, p<0.01; ***, p<0.001. ESCC, esophageal squamous cell carcinoma; KEGG, kyoto encyclopedia of genes and genomes; ns, p≥0.05; **, p<0.01; ***, p<0.001.

All ESCC biopsies were subjected to RNA sequencing (RNA-Seq). Unsupervised clustering by the euclidean method within the ConsensusClusterPlus function based on immune infiltration was used to classify the 23 ESCC patients into two clusters, the immune-hot group and the immune-cold group (**Figure 2A**). By comparing the differences in the immune microenvironment between immune-hot and immune-cold tumors, we found that tumors from the immune-hot group were highly infiltrated with B-cells, macrophages, CD45, CD8+ T-cells, cytotoxic cells, neutrophils, NK cells, T-cells, and Th1 cells (**Figure 2B**, **Supplementary Figure S3**). To validate the stability of the results, we extracted RNA-seq data from 96 esophagus cancer (ESCA) samples in the TCGA database and performed a comparison of the immune microenvironment between immune-hot and immune-cold tumors, which yielded similar results. In the ESCA analysis, hot tumors also exhibited high infiltration of CD8+ T-cells, macrophages, and NK cells (**Supplementary Figure S4**).

In our study, Differential gene expression analysis was performed on the raw expression counts of all genes between immune-cold group and immune-hot group. Thresholds for the adjust p-value (padj) and |log2FC| were set at <0.05 and ≥1, respectively. As a result, a volcano plot revealed 382 genes significantly upregulated in immune-hot group tumors, and 446 genes significantly upregulated in immune-cold tumors, with adjusted p-values less than 0.05 (**Figure 2C**). We next compared the mRNA expression profiles and signal pathway enrichment between the two groups. Olfactory transduction, Cytokine−cytokine receptor interaction, Neuroactive ligand−receptor interaction and JAK-STAT signals were significantly enriched in the immune cold group (**Figure 2D**). GSEA enrichment analysis was conducted to identify pathways upregulated in cold and hot tumors. Pathways related to the detection of stimulus (NES = 1.48, padj = 2.2 x 10-2) were upregulated in the immune-hot tumors (**Supplementary Table S1**).

**Figure 3:**
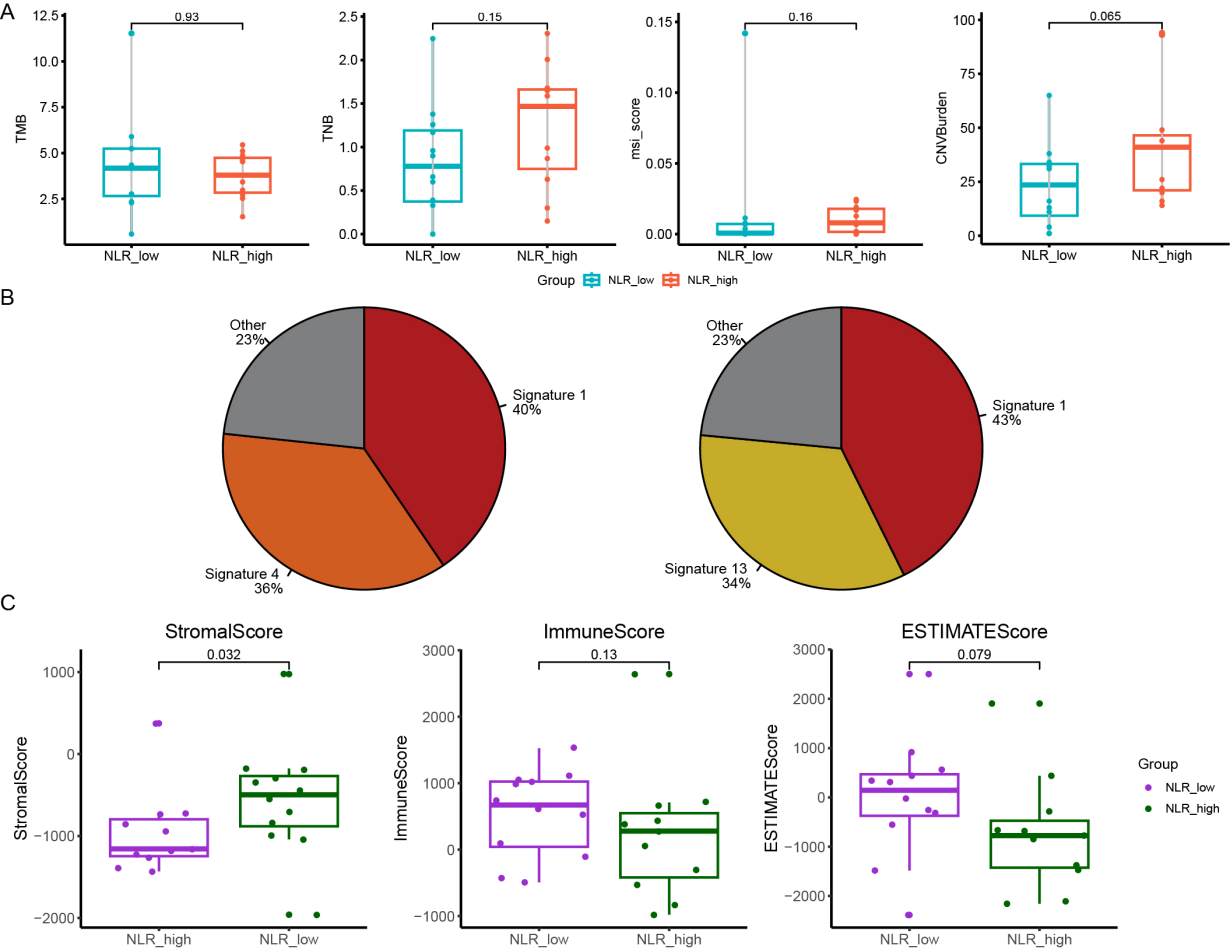
The relationship between genomic features and PFS (progression free survival) and NLR (neutrophil-to-lymphocyte ratio). **(A)** The difference in the median TMB, TNB, the chromosomal CNV burden and MSI-score between PFS-low group and PFS-high group; **(B)** The Catalogue of Somatic Mutations in Cancer (COSMIC) mutational signatures composition in patients of PFS-low group (right panel) and PFS-high group (left panel); **(C)** The differences of the ImmuneScore, StromalScore and ESTIMATEScore in NLR-high group and NLR-low group. PFS, progression free survival; NLR, neutrophil-to-lymphocyte ratio; TMB, tumor mutation burden; TNB, tumor neoantigen burden; CNV, copy number variant; MSI, microsatellite instability.

To explore the relationship between genomic and transcription characteristics and prognosis, we divided patients into two groups: PFS-low group (PFS ≤ 14m, n=12) and PFS-high group (PFS > 14m, n=11). Fisher’s exact test was used to find the differentially mutated genes between the PFS-low group and the PFS-high group. The result was not significant. We found the median TMB in the PFS-low group was significantly higher (p=0.049). However, no significant difference in TNB (p=0.15), chromosomal CNV burden (p=0.065), and MSI score (p=0.16) between two groups (**Figure 3A**). The composition of COSMIC mutational signatures was also compared between the PFS-high group and the PFS-low group (**Figure 3B**). Mutational signature 1 was present in both the PFS-high group and the PFS-low group and it was the highest in both groups. The PFS-high group was mainly associated with signature 1 (40%) and signature 4 [36%, exposure to tobacco (smoking) mutagens], and the PFS-low group were a signature 1 (43%) and signature 13 [34%, APOBEC Cytidine Deaminase (C>G)] (**Figure 3B**). The analysis suggests that patients with a better prognosis tend to have a lower TMB.

Based on RNA-seq transcriptome data, we next compared the pattern of gene expression between PFS-high group and PFS-low group using the Expression data (ESTIMATE) algorithm to calculate the ImmuneScore, StromalScore and ESTIMATEScore. We found no differences in these areas between the two groups (**Supplementary Figure S5A**). We then assessed differences in immune cell composition between two groups and found no difference in the ratio of 22 immune cells (**Supplementary Figure S5B**). This suggests that the immune composition may not differ significantly between patients with different prognosis. Different immune microenvironment may have limited influence on prognosis of ESCC.

We next used the systemic inflammation biomarker, NLR, to stratify patients and further analyze differences in genomic and transcription characteristics between different NLRs. We divided patients into two groups: the NLR-low group (0.93≤NLR ≤ 1.79, n=12) and the NLR-high group (1.93≤NLR ≤ 8.47, n=11). *KMT2D* was mutated in 5 samples (5/12) of the NLR-low group and no mutation was detected in the NLR-high Group (**Supplementary Figure S6A**). Meantimes, we found *KMT2D* mutant samples tended to have higher TMB but no significant difference (**Supplementary Figure S6B**). No significant difference in TMB, TNB, and chromosomal CNV burden between NLR-low group and NLR-high group (p = 0.93, p = 0.15 and p = 0.065, respectively) (**Supplementary Figure S6C**). The composition of COSMIC mutational signatures was also compared between the NLR-high group and the NLR-low group. The NLR-high group was mainly associated with signature 1 (62%), and NLR- low group was signature 1 (43%) and 2 (30%) (**Supplementary Figure S6D** **).**

In addition, we calculated the ImmuneScore, StromalScore, and ESTIMATEScore comparing the NLR-high group and the NLR-low group. StromalScore in the NLR-low group was significantly higher than that in the NLR-high group (p = 0.032) (**Figure 3C**). There was no significant difference in the ESTIMATEScore and ImmuneScore between two groups (p = 0.079, p = 0.13) (**Figure 3C**). The proportion of 22 kinds of immune cells in the two groups was analyzed. It was found that the proportion of B_cells_naive and T_cells_CD4_memory_activated in the NLR-low group was significantly higher than that in the NLR-high group, and there was no significant difference in other immune cells (**Supplementary Figure S7**). This suggests that naïve B cells and activated CD4+ memory T cells and NLR may have some correlation with NLR levels (**Supplementary Table S2**).

### 3.4 Differences of TCR repertoires during radiotherapy

**Figure 4:**
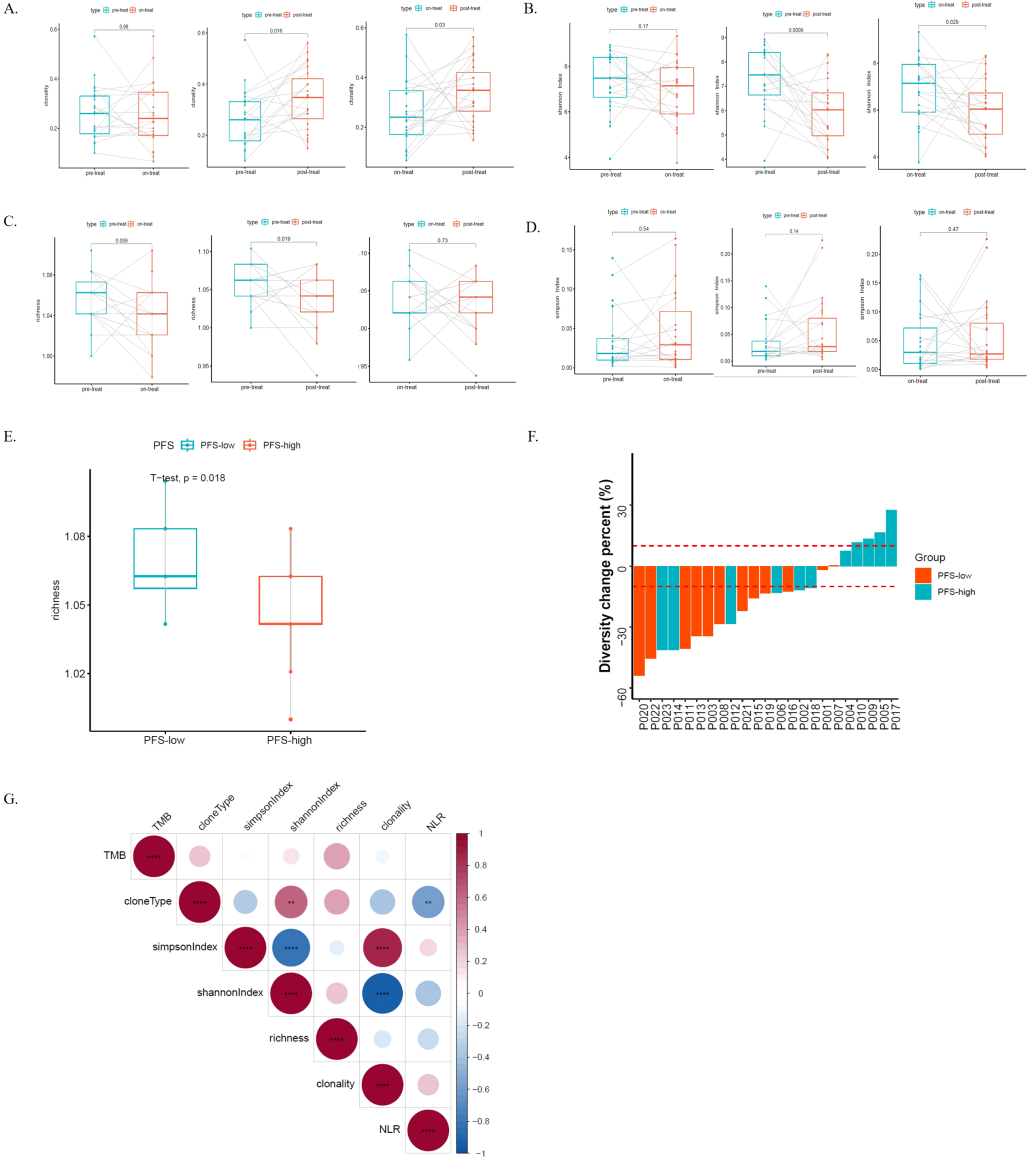
TCRs dynamics and its genomic and transcriptome association during the radio-chemotherapy of ESCC. **(A)** The TCR clonality among the pre-treatment (pre-treat), on-treatment (on-treat) and post-treatment (post-treat) ESCC patients; **(B)** The shannon index among the pre-treat, on-treat and post-treat ESCC patients; **(C)** The richness among the pre-treat, on-treat and post-treat ESCC patients; **(D)** The simpson Index among the pre-treat, on-treat and post-treat ESCC patients; **(E)** The differences of the richness of the PFS-low groups and PFS-high groups in the pre-treat sample; **(F)** The TCR-Diversity change percent in the PFS-low groups and PFS-high groups; **(G)** The correlation analysis of different indicators, including TMB, cloneType, NLR, clonality, simpson Index, shannon index and richness. The asterisks denote the significance thresholds set based on p-values **, p < 0.01; ****, p < 0.0001. Blue indicates negative correlation, while red indicates positive correlation. Correlation scores were obtained through Spearman correlation. TCR, T-cell receptors; ESCC, esophageal squamous cell carcinoma; pre-treat, pre-treatment; on-treat, on-treatment; post-treat, post-treatment; PFS, progression free survival; NLR, neutrophil-to-lymphocyte ratio; TMB, tumor mutation burden.

We compared TCR clonality, Simpson index (a type of diversity index, the probability that two clones randomly sampled belong to the same population, and the lower the Simpson index value, the higher the diversity), Shannon index (a type of diversity index, and the lower the Shannon index value, the higher the clonal diversity), and richness among the pre-treatment (pre-treat), on-treatment (on-treat) and post-treatment (post-treat) of ESCC patients by t test using matched samples. Clonality was significantly increased from pre-treat to post-treat (p = 0.016) and from on-treat to post-treat (p = 0.03) (**Figure 4A**). Shannon index was significantly decreased from pre-treat to post-treat (p = 0.0009) and from on-treat to post-treat (p = 0.029) (**Figure 4B**). Richness was significantly decreased from pre-treat to post-treat (p = 0.019) (**Figure 4C**). There was no significant difference in the Simpson index among the pre-treat, on-treat, and post-treat (**Figure 4D**). Two patients exhibited a continuous increase of TCR CDR3 (CHCLPAED_AGGGELFF) frequency in post-treatment samples compared to on-treatment samples. Three patients had elevated levels of CDR3 amino acid sequence (CASSLDSNQPQHF) after treatment. The lower the Shannon Index, the higher the diversity of TCR, and the diversity of TCR decreased after treatment. This suggests that radiotherapy in ESCC patients may result in reduced TCR diversity. To further validate our findings, we performed a similar analysis in the GSE120101 dataset, which showed comparable results. Specifically, Clonality and Simpson index exhibited an upward trend post-treatment, while Shannon index and Richness both decreased from pre-treatment to post-treatment, consistent with our study’s findings (**Supplementary Figure S8**).

We assessed the relationship between CDR3 diversity and clinical and molecular characteristics in ESCC patients. As immune status can be reflected by TCR diversity, we first evaluated TCR Clonality, Simpson index, Shannon index, and Richness differences between patients with different NLRs during the course of treatment to further investigate how TCR diversity in peripheral blood reflects immune status in ESCC. Unfortunately, there was no difference between the NLR-high group and the NLR-low group in the pre-treat, on-treat, or post-treat samples (**Supplementary Figure S9**). We continued to explore the prognostic value of the TCR repertoire for ESCC patient outcomes post-radiotherapy. To further demonstrate the similarity between the PFS-high and the PFS-low group, we focused on diversity in the Clonality, Simpson Index, Shannon index, and richness analysis, for each patient were analyzed during treatment. We found that the richness of PFS-low groups was significantly higher than that of PFS-high groups in the pre-treat sample (p = 0.018) (**Figure 4E**), while there was no significant difference in the on-treat and post-treat samples (**Supplementary Figure S10**). There was no significant difference in the Clonality, Simpson index, and Shannon index between the PFS-high and the PFS-low groups (**Supplementary Figure S10**). Subsequently, the changes in the TCR Diversity of 23 ESCC patients were analyzed. The results showed that patients with a significant increase in TCR-Diversity had better PFS and sustained clinical benefit in the PFS-high group (p = 0.024) (**Figure 4F**). In the correlation analysis, we found that clone type was significantly negatively correlated with NLR (p < 0.01), and the Shannon index was negatively correlated with NLR (**Figure 4G**).

To examine the effect of gene mutations on the TCR clonal pattern, we assessed the clonal differences between the wild-type and mutant variants, employing an unpaired t-test for statistical comparison. We found that the baseline clonality in mutant *FBXW7* (MT) was significantly lower than that in wild-type (WT) patients (p = 0.0076), while they were not significant after treatment between *FBXW7*-MT and WT patients (p = 0.059 in on-treatment samples; p = 0.18 in post-treat samples) (**Supplementary Figure S11**). Intriguingly, in *FBXW7*-MT patients, the Clonality that underwent therapy was higher than that at baseline. During radio-chemotherapy, the clonality in *RYR1*-MT and *UNC79*-MT patients had a similar phenomenon to the *FBXW7*-MT patients. The baseline clonality in *RYR1*-MT and *UNC79*-MT were significantly lower than that in WT patients (p = 0.0073 for *RYR1* and p = 0.033 for *UNC79*), while they were not significant after treatment between MT and WT patients (**Supplementary Figure S11**). The results suggested that *FBXW7*, *RYR1*, and *UNC79* mutant patients might have better treatment outcomes upon radio-chemotherapy.

## 4 Discussion

ESCC is the most common histological subtype of EC, which is a life-threatening thoracic tumor with a poor prognosis (4). Therefore, it is necessary to find molecular factors related to radical radiotherapy and chemotherapy response of ESCC. High-throughput sequencing techniques provide us with a means to search for molecular features. With its rapid development and widespread application, we have better understanding of tumor development and progression from the molecular perspective, which has had a profound effect on clinical treatment modes and survival outcomes of patients with various of cancers (36, 37). DNA and RNA-based NGS have also been utilized in supporting treatment decisions for cancer patients, early diagnosis and screening, and tumor progression (36–38). In this study, we employed whole exome sequencing (WES) to reveal the genomic landscape of ESCC, immune microenvironment characteristics were performed by whole transcriptome sequencing (WTS), and we also analyzed the relationship between genomic and immune microenvironment characteristics and prognosis of ESCCs treated with radiotherapy was analyzed. In addition, the dynamic TCR repertoire sequencing monitoring during radiotherapy was compared, and the correlation between the TCR repertoire and clinical characteristics and genomic features was explored. To our knowledge, this is the first study to comprehensively investigate the molecular and immune microenvironment characteristics in ESCC treated with radiotherapy using multi-omics techniques, which expands our understanding of ESCC.

In multiple previous studies, ESCC genomes were mainly characterized by hundreds of somatic mutations, copy number variation (CNV), and high frequencies of *TP53* mutations (11, 39, 40). Many important mutated genes, including *TP53*, *PIK3CA*, *NOTCH1*, *FAT1*, *FAT2*, *ZNF750*, and *KMT2D* have been identified in Chinese populations (11, 39, 40). Similarly, our study revealed that the genetic variants in ESCC were dispersedly distributed. In terms of mutation type, SNVs, and Indels were atypical events, whereas CNVs, especially amplification, were common in ESCC. Other than that, our study found that ESCC had an extremely high level of TMB and a relatively high level of chromosomal CNV burden. Consistent with the previous study, the *TP53*, RTK/RAS, and NOTCH pathways were concurrently prevalent in ESCC in this study. Moreover, this study suggested that some less prevalent pathways, including WNT and HIPPO pathways, also exhibited superior frequencies in ESCC (35). WNT pathway was commonly altered regardless of MMR status and the HIPPO pathway components are structurally and functionally conserved and are notable for their role in controlling organ size (41, 42). Among the distinct pathways, multiple RTK/RAS alterations and WNT alterations tended to be concurrent in one patient. Whether these variations in the multiple signaling pathways all contribute to ESCC remains to be explored.

Meanwhile, our results also showed the immune-cold group was significantly enriched in JAK-STAT signaling pathway. Previous studies have found that activation of the JAK-STAT signaling pathway is associated with cell proliferation and metastasis in EC (43, 44). Meantime, we have found the detection of stimulus pathways (NES=1.48, p=2.2 x 10-2) were upregulated in the immune-hot tumors by GSEA enrichment analysis. The stimulus pathways involved in the perception of pain in which a stimulus is received and converted into a molecular signal. Previous studies have reported that methylation gene analysis in thyroid cancer is enriched in pathways related to the detection of stimulus, but no further analysis has been conducted (45). Also, this pathway was reported in 2023, suggesting that the down-regulated genes are highly involved in retinal function and homeostasis (46). However, the study of the ESCC immune-hot group and this signaling pathway has not been reported, and the specific biological mechanism need to be further studied.

Previous studies have found hot tumor immune status was not associated with poor prognosis compared to the other groups in ESCC (47). A study found IS (immune subtype) 1 can be considered “hot,” with high immune infiltrate respond better to immunotherapy and mRNA vaccines, while IS2 patients respond less well to immune-related treatments in ESCC (48). Meantime, ESCC samples were divided into three ICI (immune cell infiltration) types that may help guide immunotherapy in the future, because ICI cluster B presented an immuno-activated phenotype with high immune, and this was accompanied by high levels of CD8 T cells, activated memory CD4 T cells, and activated NK cells. Activated NK cells were consistent with this study, but CD8 T cells, and activated memory CD4 T cells showed different results (49). Among other cell types, Mast cells resting appears significantly in immune-hot groups. Previous studies on cell types in normal and tumor tissue of ESCC have reported that Activated memory CD4 T cells, M0 macrophages, M1 macrophages, and Neutrophils are significantly found in the tumor tissue, while Eosinophils, Resting mast cells, Monocytes, Gamma delta T cells, Regulatory T cells (Tregs), Plasma cells and Memory B cells were significantly enriched in normal tissue. Activated NK cells were highly expressed in normal tissues without significant differences (50). Meantime, higher proportions of resting memory CD4 and Gamma delta T cells, in addition to M0 and M2 macrophages, were also found to be negative prognostic markers of clinical outcome. In contrast, greater infiltration of plasma cells, CD8 T cells, activated NK cells, and resting mast cells was correlated with improved prognosis (51). In this study, the immune-hot group were highly infiltrated with B-cells, macrophages, CD45, CD8+ T-cells, cytotoxic cells, neutrophils, NK cells, T-cells, and Th1 cells. TCGA-ESCA database validation analysis of the immune microenvironment between immune-hot and immune-cold tumors in the TCGA-ESCA database yielded similar results.

TMB, which represents the number of mutations per megabase of sequenced DNA in cancer, has been demonstrated to be a biomarker for immune checkpoint inhibitors across some cancer types (52, 53). TMB values vary widely among pan-solid tumors (54), and the prognostic value of TMB in patients with solid tumors is controversial (53). A previous study observed the relationship between TMB and prognosis, high TMB had a poor prognosis in all cohorts, but in 90 WES samples, high TMB had a good prognosis (40). A previous study showed that the TMB was not significantly correlated with the response to radiotherapy in ESCC patients (55). And then in our study, the median TMB in the PFS-low group was significantly higher than that in the PFS-high group and no significant difference in the ratio of immune cells between the two groups, which suggests that patients with a better prognosis tend to have a lower tumor molecular burden and different immune microenvironment may have limited influence on prognosis of ESCC. However, as the study sample size is small, or as the problem of technical resolution, and whether the analysis of single-cell transcriptome based on a more detailed immune cell population can solve this problem, further prospective validation studies are required.

NLR is a peripheral blood biomarker, whose alterations are capable of representing systemic inflammation in patients (56). In EC, NLR is associated with tumor progression and is predictive of poorer survival in patients (57). NLR is a predictor of the response to immune checkpoint inhibitor treatment in patients with ESCC, and the PFS rate in ESCC patients with low NLR (Post treatment, at 6 weeks) was higher than in patients with high NLR (P = 0.027). In other scenarios, the difference was not statistically significant including baseline low NLR (58). Our study explored the immune cell composition and immune microenvironment in tumor tissues from different NLR patients, intending to be able to analyze the response to factors that may influence the immune checkpoint inhibitor treatment and prognosis. It was found that the proportion of naive B cells and activated T cells CD4 memory in NLR-low group was significantly higher than that in the NLR-high group, and the NLR-low group had significantly higher StromalScore than the NLR-high group, and the NLR-low group ESTIMATE is higher than the NLR-high group, but the difference is not significant. When StromalScore and ESTIMATE are high, it may indicate that there are more stromal components in tumor tissue and the infiltration of immune cells is significant. This may be associated with increased tumor infiltration, immune cell activity, or other microenvironmental factors. *KMT2D* has been reported to play an anti-cancer role in ESCC (40). The previous study discovered that the *KMT2D* was associated with the multiple clinical characteristics of ESCC and its expression in tumor tissue is lower than that in normal tissue (40). In our cohort, it seemed that the incidence of *KMT2D* mutations was lower in the NLR-low group than in the NLR-high group. Patients with low NLR values had a high incidence of *KMT2D* mutations, which required to be verified in further studies.

In our study, we found that the baseline clonality in *FBXW7*, *RYR1*, and *UNC79* mutant patients was lower than that in wild patients, while they were not significant after treatment. In mutant patients, the clonality that underwent therapy was higher than that at baseline. Previous research reports that the *FBXW7* gene is a p53-dependent tumor suppressor gene, which targets mTOR for degradation and cooperates with *PTEN* in tumor suppression. Loss or mutation of *FBXW7* makes the tumor cells sensitive to treatment (59). RYR1 is a subtype of RYRs, and the alterations of RYRs play key roles in a series of rare diseases (60). The *RYR1* gene which is fundamental to the process of excitation-contraction coupling and skeletal muscle calcium homeostasis, is associated with proliferation and apoptosis of various tumors (60, 61). *UNC79* genes encoding UNC-79 proteins may be susceptibility loci for several diseases including cancer (62). The *FBXW7*, *RYR1*, and *UNC79* gene mutations may play a role in tumorigenesis and development (62–65). The results of our study suggested that the *FBXW7*, *RYR1*, and *UNC79* mutant patients might be sensitive to treatment and *FBXW7*, *RYR1*, and *UNC79* might contribute to immune response upon radio-chemotherapy.

In addition, we observed a significant increase in clonality from pre-treatment to post-treatment (p = 0.016) as well as from mid-treatment to post-treatment (p = 0.03). Meanwhile, the Shannon index showed a notable decrease from pre-treatment to post-treatment (p = 0.0009) and from mid-treatment to post-treatment (p = 0.029), indicating a reduction in TCR diversity after treatment. This suggests that radiation therapy in ESCC patients might lead to decreased TCR diversity and increased clonality. TCR clonality is associated with treatment in multiple studies. Hopkins et al.’s study demonstrated the association between TCR diversity, T-cell clonal changes, and immunotherapeutic efficacy in pancreatic cancer. They found that patients with higher pre-treatment TCR diversity or post-treatment clonal expansion had longer survival rates (66). Ford et al.’s 2018 study revealed that patients who received neoadjuvant therapy and achieved significant pathological responses exhibited higher TCR clonality (67). Our study further clarified the relationship between TCR clonality and chemoradiotherapy. Additionally, we observed that in pre-treatment samples, patients with shorter PFS had significantly higher clone abundance compared to those with longer PFS (p = 0.018), suggesting fewer clone types in patients with longer PFS. This contrasts with previous conclusions; Benjamin A. Kansy et al. showed an association between increased TCR sequence abundance and improved treatment response (p = 0.03) (68), while S. Ji et al. found significantly higher disease control rates in patients with high baseline TCR diversity (69). Clonality was significantly increased from pre-treatment to post-treatment (p = 0.016) and from on-treatment to post-treatment (p = 0.03). Increased clonality after treatment may be associated with better survival or PFS. Shannon index was significantly decreased from pre-treatment to post-treatment (p = 0.0009) and from on-treatment to post-treatment (p = 0.029). The lower the Shannon Index, the higher the diversity of TCR, and the diversity of TCR decreased after treatment. This suggests that radiotherapy in ESCC patients may result in reduced TCR diversity. We found that the richness of PFS-low groups was significantly higher than that of PFS-high groups in the pre-treat sample (p = 0.018). This suggests that patients with longer PFS have fewer clonal types.

However, there are still some limitations in this study. Firstly, this study was the small amount number of participants, which may reduce the representativeness of certain findings, particularly in a disease as heterogeneous as ESCC. Therefore, subsequent studies with large sample size are still needed to verify the results of this study. Secondly, our study primarily provides cross-sectional data, longitudinal RNA sequencing and overall survival (OS) were absent, and that was a non-immunotherapy cohort. Therefore, there are deficiencies in a more in-depth analysis of the dynamics of immune response and the change of treatment effect over time, and there is no gold standard comparison of OS. Follow-up studies should focus on an in-depth analysis of molecular dynamic response in immunotherapy and the relationship between multiple omics at different nodes, and *in vitro* validation of the obtained conclusions should be carried out under necessary conditions to improve the overall research depth. Third, we only conducted validation of external ESCA RNA data and advanced solid tumors TCR data (**Supplementary Figures S4**, **S8**), and the validation results were consistent with the present study. We still lack a multi-node, multi-omics external validation queue to adequately explain our findings. Therefore, in the follow-up study, it is necessary to increase the sample size and set the independent verification cohort to improve the statistical robustness of conclusions.

In conclusion, multi-omics sequencing techniques help us better understand the molecular characteristics of ESCC. Based on the genomic and transcriptomic analysis, we can identify potential biomarkers of ESCC, especially immune microenvironment characteristics. Based on TCR clonality and Shannon index analysis among the pre-treatment, on-treatment, and post-treatment ESCC patients using paired samples, we concluded that TCRs are clonal expansion after radiotherapy and chemotherapy in ESCC, suggesting an immune-activated microenvironment after radio-chemotherapy. Our multi-omics analysis provided the basic TCRs dynamics and its genomic and transcriptome association during the radio-chemotherapy of EC, which may provide new ideas for the diagnosis and treatment of ESCC.

## Data Availability

The data presented in the study are deposited in the Genome Sequence Archive (GSA-Human) database. The accession number is HRA009574.

## Supplementary material

Supplementary Figure 1 Mutation signatures in ESCC samples. **(A)** Contributions of six possible substitution types at different nucleotide contexts; **(B)** The Catalogue of Somatic Mutations in Cancer (COSMIC) mutational signatures composition in 23 ESCC patients. ESCC, esophageal squamous cell carcinoma.

Supplementary Figure 2 The TMB of the 23 ESCC patients in this study and other cancers derived from TCGA. The numerical values above the figure indicate the total sample size for each cancer cohort. ESCC, esophageal squamous cell carcinoma; TMB, tumor mutation burden.

Supplementary Figure 3 Differential expression analysis of immune cell between PFS-H and PFS-L groups using multiple methodologies. ns, p≥0.05; **, p<0.01; ***, p<0.001.

Supplementary Figure 4 The association of the immune infiltration derived from TCGA-ESCA database RNA sequencing. ns, p≥0.05; *, p<0.05; **, p<0.01; ***, p<0.001; ****, p < 0.0001.

Supplementary Figure 5 The Immune-Score and the ratio of immune cells in PFS-high group and PFS-low group. **(A)** the ImmuneScore, StromalScore and ESTIMATEScore, in PFS-high group and PFS-low group; **(B)** The ratio of 22 immune cells between PFS-high group and PFS-low group. The numbers in the boxplots represent the median scores of each group. PFS, progression free survival.

Supplementary Figure 6 The association of genomic characteristics between different NLRs (neutrophil-to-lymphocyte ratio). **(A)** The incidence of mutations in NLR-high group and NLR-low group; **(B)** The association between mutated gene and tumor mutational burden, *KMT2D* mutant (*KMT2D*-mut) samples tended to have higher TMB than *KMT2D* wild-type (*KMT2D*- wild) samples; **(C)** The difference in the median TMB, TNB and the chromosomal CNV burden between NLR-high group and NLR-low group. The numbers in the figure represent the p-values obtained from the Wilcoxon rank-sum test. **(D)** The Catalogue of Somatic Mutations in Cancer (COSMIC) mutational signatures composition in patients of NLR-low group (left panel) and NLR-high group (right panel). NLR, neutrophil-to-lymphocyte ratio; TMB, tumor mutation burden; TNB, tumor neoantigen burden; CNV, copy number variant.

Supplementary Figure 7 The differences of the composition of immune cells between NLR-high group and NLR-low group. NLR, neutrophil-to-lymphocyte ratio; ns, p≥0.05; **, p<0.01; ***, p<0.001.

Supplementary Figure 8 TCRs dynamics verification by an external GSE120101 dataset of solid tumors.

Supplementary Figure 9 The differences of the TCR clonality, simpson Index, shannon index and richness between NLR-high group and NLR-low group in the pre-treat, on-treat, or post-treat samples. TCR, T-cell receptors; NLR, neutrophil-to-lymphocyte ratio; pre-treat, pre-treatment; on-treat, on-treatment; post-treat, post-treatment.

Supplementary Figure 10 The differences of the TCR clonality, simpson Index, shannon index and richness between PFS-high group and PFS-low group in the pre-treat, on-treat, or post-treat samples. TCR, T-cell receptors; PFS, progression free survival; pre-treat, pre-treatment; on-treat, on-treatment; post-treat, post-treatment.

Supplementary Figure 11 The association between the Clonality and mutated genes.

## References

[1] Sung H Ferlay J Siegel RL Laversanne M Soerjomataram I Jemal A. Global cancer statistics 2020: GLOBOCAN estimates of incidence and mortality worldwide for 36 cancers in 185 countries. CA Cancer J Clin. (2021) 71:209–49. doi: 10.3322/caac.21660 33538338

[2] Qiu H Cao S Xu R. Cancer incidence, mortality, and burden in China: a time-trend analysis and comparison with the United States and United Kingdom based on the global epidemiological data released in 2020. Cancer Commun (London England). (2021) 41:1037–48. doi: 10.1002/cac2.12197 PMC8504144 34288593

[3] Arnold M Soerjomataram I Ferlay J Forman D. Global incidence of oesophageal cancer by histological subtype in 2012. Gut. (2015) 64:381–7. doi: 10.1136/gutjnl-2014-308124 25320104

[4] Businello G Parente P Mastracci L Pennelli G Traverso G Milione M. The pathologic and molecular landscape of esophageal squamous cell carcinogenesis. Cancers (Basel). (2020) 12(8):2160. doi: 10.3390/cancers12082160 32759723 PMC7465394

[5] Cao W Chen HD Yu YW Li N Chen WQ. Changing profiles of cancer burden worldwide and in China: a secondary analysis of the global cancer statistics 2020. Chin Med J. (2021) 134:783–91. doi: 10.1097/cm9.0000000000001474 PMC8104205 33734139

[6] Waters JK Reznik SI. Update on management of squamous cell esophageal cancer. Curr Oncol Rep. (2022) 24:375–85. doi: 10.1007/s11912-021-01153-4 35142974

[7] Nobel TB Barbetta A Hsu M Tan KS Pinchinat T Schlottmann F. Outcomes of radiation-associated esophageal squamous cell carcinoma: the MSKCC experience. J gastrointestinal surgery: Off J Soc Surg Alimentary Tract. (2019) 23:11–22. doi: 10.1007/s11605-018-3958-8 PMC6572721 30215197

[8] Codipilly DC Wang KK. Squamous cell carcinoma of the esophagus. Gastroenterol Clinics North America. (2022) 51:457–84. doi: 10.1016/j.gtc.2022.06.005 36153105

[9] Weidenbaum C Gibson MK. Approach to localized squamous cell cancer of the esophagus. Curr Treat options Oncol. (2022) 23:1370–87. doi: 10.1007/s11864-022-01003-w PMC9526684 36042147

[10] Vasaikar SV Straub P Wang J Zhang B. LinkedOmics: analyzing multi-omics data within and across 32 cancer types. Nucleic Acids Res. (2018) 46(D1):D956–d63. doi: 10.1093/nar/gkx1090 PMC5753188 29136207

[11] Liu Z Zhao Y Kong P Liu Y Huang J Xu E. Integrated multi-omics profiling yields a clinically relevant molecular classification for esophageal squamous cell carcinoma. Cancer Cell. (2023) 41:181–95.e9. doi: 10.1016/j.ccell.2022.12.004 36584672

[12] Cui Y Chen H Xi R Cui H Zhao Y Xu E. Whole-genome sequencing of 508 patients identifies key molecular features associated with poor prognosis in esophageal squamous cell carcinoma. Cell Res. (2020) 30:902–13. doi: 10.1038/s41422-020-0333-6 PMC7608103 32398863

[13] Cao W Lee H Wu W Zaman A McCorkle S Yan M. Multi-faceted epigenetic dysregulation of gene expression promotes esophageal squamous cell carcinoma. Nat Commun. (2020) 11:3675. doi: 10.1038/s41467-020-17227-z 32699215 PMC7376194

[14] Yang H Wang Y Jia Z Wang Y Yang X Wu P. Characteristics of T-cell receptor repertoire and correlation with EGFR mutations in all stages of lung cancer. Front Oncol. (2021) 11:537735. doi: 10.3389/fonc.2021.537735 33777727 PMC7991722

[15] Hsu M Sedighim S Wang T Antonios JP Everson RG Tucker AM. TCR sequencing can identify and track glioma-infiltrating T cells after DC vaccination. Cancer Immunol Res. (2016) 4:412–18. doi: 10.1158/2326-6066.cir-15-0240 PMC4873445 26968205

[16] Han J Duan J Bai H Wang Y Wan R Wang X. TCR repertoire diversity of peripheral PD-1(+)CD8(+) T cells predicts clinical outcomes after immunotherapy in patients with non-small cell lung cancer. Cancer Immunol Res. (2020) 8:146–54. doi: 10.1158/2326-6066.cir-19-0398 31719056

[17] Liu Y Lu T Yuan M Chen R Lu J Wang H. Genomic and transcriptomic insights into the precision treatment of pulmonary enteric adenocarcinoma. Lung Cancer (Amsterdam Netherlands). (2023) 179:107169. doi: 10.1016/j.lungcan.2023.03.005 37003209

[18] Wu H Yu Z Liu Y Guo L Teng L Guo L. Genomic characterization reveals distinct mutation landscapes and therapeutic implications in neuroendocrine carcinomas of the gastrointestinal tract. Cancer Commun (London England). (2022) 42:1367–86. doi: 10.1002/cac2.12372 PMC9759768 36264285

[19] Bolotin DA Poslavsky S Mitrophanov I Shugay M Mamedov IZ Putintseva EV. MiXCR: software for comprehensive adaptive immunity profiling. Nat Methods. (2015) 12:380–1. doi: 10.1038/nmeth.3364 25924071

[20] Chen S Zhou Y Chen Y Gu J. fastp: an ultra-fast all-in-one FASTQ preprocessor. Bioinf (Oxford England). (2018) 34:i884–i90. doi: 10.1093/bioinformatics/bty560 PMC6129281 30423086

[21] Li H Durbin R. Fast and accurate short read alignment with Burrows-Wheeler transform. Bioinf (Oxford England). (2009) 25:1754–60. doi: 10.1093/bioinformatics/btp324 PMC2705234 19451168

[22] Kim D Langmead B Salzberg SL. HISAT: a fast spliced aligner with low memory requirements. Nat Methods. (2015) 12:357–60. doi: 10.1038/nmeth.3317 PMC4655817 25751142

[23] Cibulskis K Lawrence MS Carter SL Sivachenko A Jaffe D Sougnez C. Sensitive detection of somatic point mutations in impure and heterogeneous cancer samples. Nat Biotechnol. (2013) 31:213–9. doi: 10.1038/nbt.2514 PMC3833702 23396013

[24] Li J Lupat R Amarasinghe KC Thompson ER Doyle MA Ryland GL. CONTRA: copy number analysis for targeted resequencing. Bioinf (Oxford England). (2012) 28:1307–13. doi: 10.1093/bioinformatics/bts146 PMC3348560 22474122

[25] Pertea M Pertea GM Antonescu CM Chang TC Mendell JT Salzberg SL. StringTie enables improved reconstruction of a transcriptome from RNA-seq reads. Nat Biotechnol. (2015) 33:290–5. doi: 10.1038/nbt.3122 PMC4643835 25690850

[26] Mermel CH Schumacher SE Hill B Meyerson ML Beroukhim R Getz G. GISTIC2.0 facilitates sensitive and confident localization of the targets of focal somatic copy-number alteration in human cancers. Genome Biol. (2011) 12:R41. doi: 10.1186/gb-2011-12-4-r41 21527027 PMC3218867

[27] Sondka Z Dhir NB Carvalho-Silva D Jupe S Madhumita McLaren K. COSMIC: a curated database of somatic variants and clinical data for cancer. Nucleic Acids Res. (2024) 52:D1210–d17. doi: 10.1093/nar/gkad986 PMC10767972 38183204

[28] Subramanian A Tamayo P Mootha VK Mukherjee S Ebert BL Gillette MA. Gene set enrichment analysis: a knowledge-based approach for interpreting genome-wide expression profiles. Proc Natl Acad Sci United States America. (2005) 102:15545–50. doi: 10.1073/pnas.0506580102 PMC1239896 16199517

[29] Kanehisa M Furumichi M Tanabe M Sato Y Morishima K. KEGG: new perspectives on genomes, pathways, diseases and drugs. Nucleic Acids Res. (2017) 45:D353–d61. doi: 10.1093/nar/gkw1092 PMC5210567 27899662

[30] Harris MA Clark J Ireland A Lomax J Ashburner M Foulger R. The Gene Ontology (GO) database and informatics resource. Nucleic Acids Res. (2004) 32:D258–61. doi: 10.1093/nar/gkh036 PMC308770 14681407

[31] Hänzelmann S Castelo R Guinney J. GSVA: gene set variation analysis for microarray and RNA-Seq data. BMC Bioinf. (2013) 14:7. doi: 10.1186/1471-2105-14-7 PMC3618321 23323831

[32] Zeng D Ye Z Shen R Yu G Wu J Xiong Y. IOBR: multi-omics immuno-oncology biological research to decode tumor microenvironment and signatures. Front Immunol. (2021) 12:687975. doi: 10.3389/fimmu.2021.687975 34276676 PMC8283787

[33] Gao YB Chen ZL Li JG Hu XD Shi XJ Sun ZM. Genetic landscape of esophageal squamous cell carcinoma. Nat Genet. (2014) 46:1097–102. doi: 10.1038/ng.3076 25151357

[34] Lin DC Hao JJ Nagata Y Xu L Shang L Meng X. Genomic and molecular characterization of esophageal squamous cell carcinoma. Nat Genet. (2014) 46:467–73. doi: 10.1038/ng.2935 PMC4070589 24686850

[35] Sanchez-Vega F Mina M Armenia J Chatila WK Luna A La KC. Oncogenic signaling pathways in the cancer genome atlas. Cell. (2018) 173:321–37.e10. doi: 10.1016/j.cell.2018.03.035 29625050 PMC6070353

[36] Miller M Hanna N. Advances in systemic therapy for non-small cell lung cancer. BMJ (Clinical Res ed). (2021) 375:n2363. doi: 10.1136/bmj.n2363 34753715

[37] Biller LH Schrag D. Diagnosis and treatment of metastatic colorectal cancer: A review. Jama. (2021) 325:669–85. doi: 10.1001/jama.2021.0106 33591350

[38] Li W Liu JB Hou LK Yu F Zhang J Wu W. Liquid biopsy in lung cancer: significance in diagnostics, prediction, and treatment monitoring. Mol Cancer. (2022) 21:25. doi: 10.1186/s12943-022-01505-z 35057806 PMC8772097

[39] Zhang N Shi J Shi X Chen W Liu J. Mutational characterization and potential prognostic biomarkers of chinese patients with esophageal squamous cell carcinoma. OncoTargets Ther. (2020) 13:12797–809. doi: 10.2147/ott.s275688 PMC7751839 33363385

[40] Zou B Guo D Kong P Wang Y Cheng X Cui Y. Integrative genomic analyses of 1,145 patient samples reveal new biomarkers in esophageal squamous cell carcinoma. Front Mol Biosci. (2021) 8:792779. doi: 10.3389/fmolb.2021.792779 35127817 PMC8814608

[41] Wang J Li R He Y Yi Y Wu H Liang Z. Next-generation sequencing reveals heterogeneous genetic alterations in key signaling pathways of mismatch repair deficient colorectal carcinomas. Modern pathology: an Off J United States Can Acad Pathology Inc. (2020) 33:2591–601. doi: 10.1038/s41379-020-0612-2 32620917

[42] Wu Z Guan KL. Hippo signaling in embryogenesis and development. Trends Biochem Sci. (2021) 46:51–63. doi: 10.1016/j.tibs.2020.08.008 32928629 PMC7749079

[43] Luo H Yang Z Zhang Q Li T Liu R Feng S. LIF inhibits proliferation of esophageal squamous carcinoma cells by radiation mediated through JAK-STAT signaling pathway. J Cancer. (2023) 14:532–43. doi: 10.7150/jca.81222 PMC10088534 37057285

[44] You Z Xu D Ji J Guo W Zhu W He J. JAK/STAT signal pathway activation promotes progression and survival of human oesophageal squamous cell carcinoma. Clin Trans oncology: Off Publ Fed Spanish Oncol Societies Natl Cancer Institute Mexico. (2012) 14:143–9. doi: 10.1007/s12094-012-0774-6 22301404

[45] Chai L Li J Lv Z. An integrated analysis of cancer genes in thyroid cancer. Oncol Rep. (2016) 35:962–70. doi: 10.3892/or.2015.4466 26718127

[46] Li L Sun Y Davis AE Shah SH Hamed LK Wu MR. Mettl14-mediated m(6)A modification ensures the cell-cycle progression of late-born retinal progenitor cells. Cell Rep. (2023) 42:112596. doi: 10.1016/j.celrep.2023.112596 37269288 PMC10543643

[47] Kuriyama K Higuchi T Yokobori T Saito H Yoshida T Hara K. Uptake of positron emission tomography tracers reflects the tumor immune status in esophageal squamous cell carcinoma. Cancer Sci. (2020) 111(6):1969–78. doi: 10.1111/cas.14421 PMC7293073 32302443

[48] Lu T Xu R Wang CH Zhao JY Peng B Wang J. Identification of tumor antigens and immune subtypes of esophageal squamous cell carcinoma for mRNA vaccine development. Front Genet. (2022) 13:853113. doi: 10.3389/fgene.2022.853113 35734437 PMC9207414

[49] Sui Z Wu X Du L Wang H Yuan L Zhang JV. Characterization of the immune cell infiltration landscape in esophageal squamous cell carcinoma. Front Oncol. (2022) 12:879326. doi: 10.3389/fonc.2022.879326 35875070 PMC9300817

[50] Li M Chen P Zhao Y Feng X Gao S Qi Y. Immune infiltration represents potential diagnostic and prognostic biomarkers for esophageal squamous cell carcinoma. BioMed Res Int. (2022) 2022:9009269. doi: 10.1155/2022/9009269 35795310 PMC9251101

[51] Yin H Wang X Jin N Ling X Leng X Wang Y. Integrated analysis of immune infiltration in esophageal carcinoma as prognostic biomarkers. Ann Transl Med. (2021) 9:1697. doi: 10.21037/atm-21-5881 34988206 PMC8667131

[52] Chan TA Yarchoan M Jaffee E Swanton C Quezada SA Stenzinger A. Development of tumor mutation burden as an immunotherapy biomarker: utility for the oncology clinic. Ann oncology: Off J Eur Soc Med Oncol. (2019) 30:44–56. doi: 10.1093/annonc/mdy495 PMC6336005 30395155

[53] Addeo A Friedlaender A Banna GL Weiss GJ. TMB or not TMB as a biomarker: That is the question. Crit Rev Oncol Hematol. (2021) 163:103374. doi: 10.1016/j.critrevonc.2021.103374 34087341

[54] Alexandrov LB Nik-Zainal S Wedge DC Aparicio SA Behjati S Biankin AV. Signatures of mutational processes in human cancer. Nature. (2013) 500:415–21. doi: 10.1038/nature12477 PMC3776390 23945592

[55] Xu X Wang Y Bai Y Lu J Guo Y Wang X. Identifying key mutations of radioresponsive genes in esophageal squamous cell carcinoma. Front Immunol. (2022) 13:1001173. doi: 10.3389/fimmu.2022.1001173 36119057 PMC9478485

[56] Cao H Shi H Zhao M Liu Z Qian J. Prognostic value of the combined preoperative plasma fibrinogen and systemic inflammatory indexes in ESCC patients. Discover Oncol. (2023) 14:143. doi: 10.1007/s12672-023-00763-7 PMC10403484 37541963

[57] Yodying H Matsuda A Miyashita M Matsumoto S Sakurazawa N Yamada M. Prognostic significance of neutrophil-to-lymphocyte ratio and platelet-to-lymphocyte ratio in oncologic outcomes of esophageal cancer: A systematic review and meta-analysis. Ann Surg Oncol. (2016) 23:646–54. doi: 10.1245/s10434-015-4869-5 26416715

[58] Wu X Han R Zhong Y Weng N Zhang A. Post treatment NLR is a predictor of response to immune checkpoint inhibitor therapy in patients with esophageal squamous cell carcinoma. Cancer Cell Int. (2021) 21:356. doi: 10.1186/s12935-021-02072-x 34233686 PMC8262036

[59] Mao JH Kim IJ Wu D Climent J Kang HC DelRosario R. FBXW7 targets mTOR for degradation and cooperates with PTEN in tumor suppression. Sci (New York NY). (2008) 321:1499–502. doi: 10.1126/science.1162981 PMC2849753 18787170

[60] Wang Y Chen Y Zhang L Xiong J Xu L Cheng C. Ryanodine receptor (RYR) mutational status correlates with tumor mutational burden, age and smoking status and stratifies non-small cell lung cancer patient prognosis. Trans Cancer Res. (2022) 11:2070–83. doi: 10.21037/tcr-21-2395 PMC9372243 35966320

[61] Robinson R Carpenter D Shaw MA Halsall J Hopkins P. Mutations in RYR1 in Malignant hyperthermia and central core disease. Hum Mutat. (2006) 27:977–89. doi: 10.1002/humu.20356 16917943

[62] Kim K Lee J Lee JY Yong SH Kim EY Jung JY. Clinical features and molecular genetics associated with brain metastasis in suspected early-stage non-small cell lung cancer. Front Oncol. (2023) 13:1148475. doi: 10.3389/fonc.2023.1148475 37139160 PMC10150586

[63] Akhoondi S Sun D von der Lehr N Apostolidou S Klotz K Maljukova A. FBXW7/hCDC4 is a general tumor suppressor in human cancer. Cancer Res. (2007) 67:9006–12. doi: 10.1158/0008-5472.can-07-1320 17909001

[64] Wang F Yu J Lin P Sigalas C Zhang S Gong Y. The ryanodine receptor mutational characteristics and its indication for cancer prognosis. Sci Rep. (2022) 12:16113. doi: 10.1038/s41598-022-19905-y 36167878 PMC9515073

[65] Iamshanova O Gordienko D Folcher A Bokhobza A Shapovalov G Kannancheri-Puthooru D. Expression of neuronal Na+ leak channel, NALCN, provides for persistent invasion of metastasizing cancer cells. bioRxiv. (2022) 2020.08.13.249169. doi: 10.1101/2020.08.13.249169

[66] Hopkins AC Yarchoan M Durham JN Yusko EC Rytlewski JA Robins HS. T cell receptor repertoire features associated with survival in immunotherapy-treated pancreatic ductal adenocarcinoma. JCI Insight. (2018) 3(13):e122092. doi: 10.1172/jci.insight.122092 29997287 PMC6124515

[67] Forde PM Chaft JE Smith KN Anagnostou V Cottrell TR Hellmann MD. Neoadjuvant PD-1 blockade in resectable lung cancer. New Engl J Med. (2018) 378:1976–86. doi: 10.1056/NEJMoa1716078 PMC6223617 29658848

[68] Kansy BA Shayan G Jie HB Gibson SP Lei YL Brandau S. T cell receptor richness in peripheral blood increases after cetuximab therapy and correlates with therapeutic response. Oncoimmunology. (2018) 7:e1494112. doi: 10.1080/2162402x.2018.1494112 30377562 PMC6205044

[69] Ji S Li J Chang L Zhao C Jia R Tan Z. Peripheral blood T-cell receptor repertoire as a predictor of clinical outcomes in gastrointestinal cancer patients treated with PD-1 inhibitor. Clin Trans oncology: Off Publ Fed Spanish Oncol Societies Natl Cancer Institute Mexico. (2021) 23:1646–56. doi: 10.1007/s12094-021-02562-4 33583004

