# Supplementary figures and images for: Genomic, transcriptomic, and T cell receptor profiling in stratifying response to first-line chemoradiotherapy or radiotherapy for esophageal squamous cell carcinoma
Source: Front Oncol. 2025 Jan 6;14:1495200. doi: 10.3389/fonc.2024.1495200 (PMC11743576; doi:10.3389/fonc.2024.1495200)

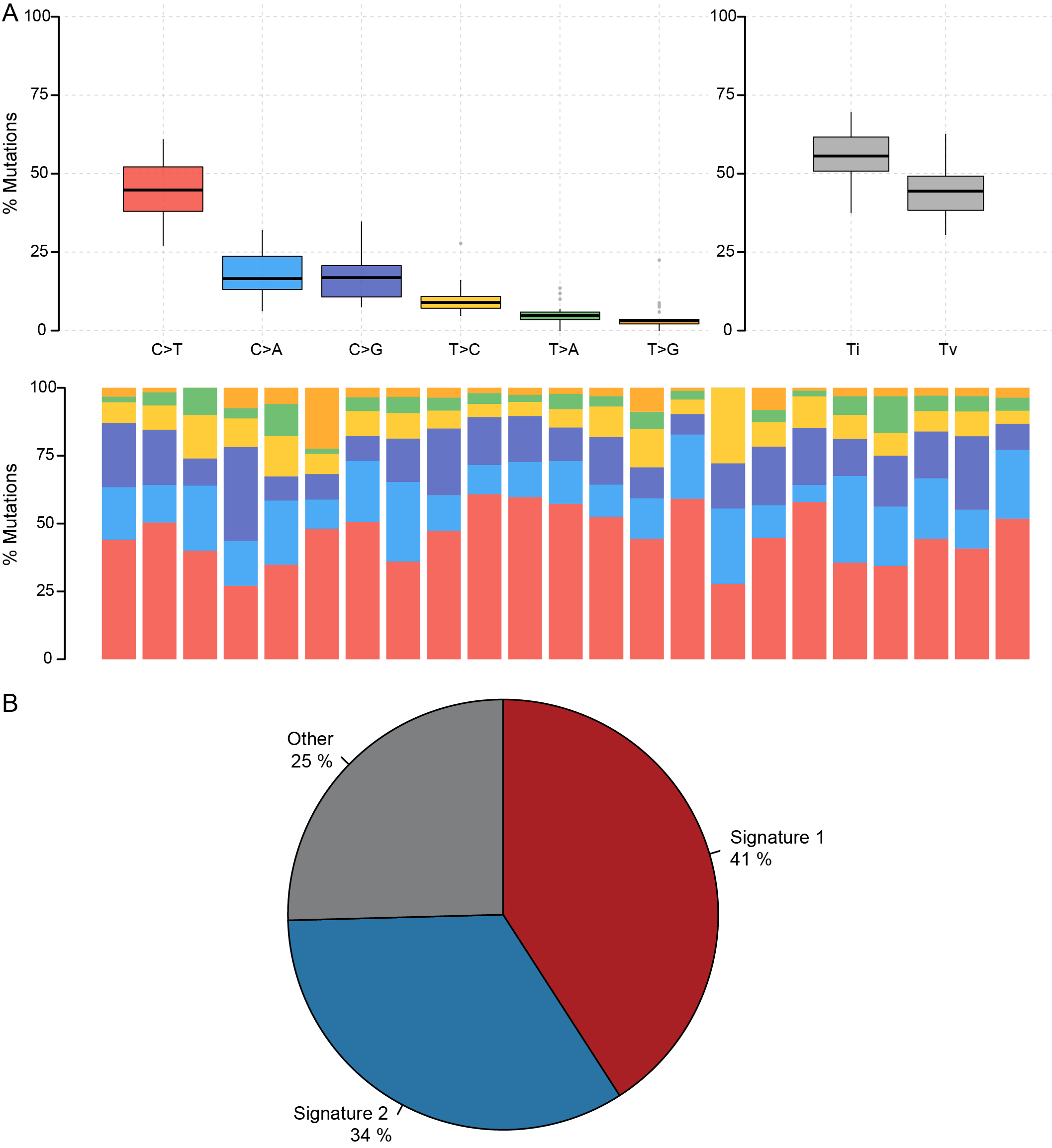

Supplement: Supplementary Figure 1 — Mutation signatures in ESCC samples. (A) Contributions of six possible substitution types at different nucleotide contexts; (B) The Catalogue of Somatic Mutations in Cancer (COSMIC) mutational signatures composition in 23 ESCC patients. ESCC, esophageal squamous cell carcinoma. [file Image1.jpeg]

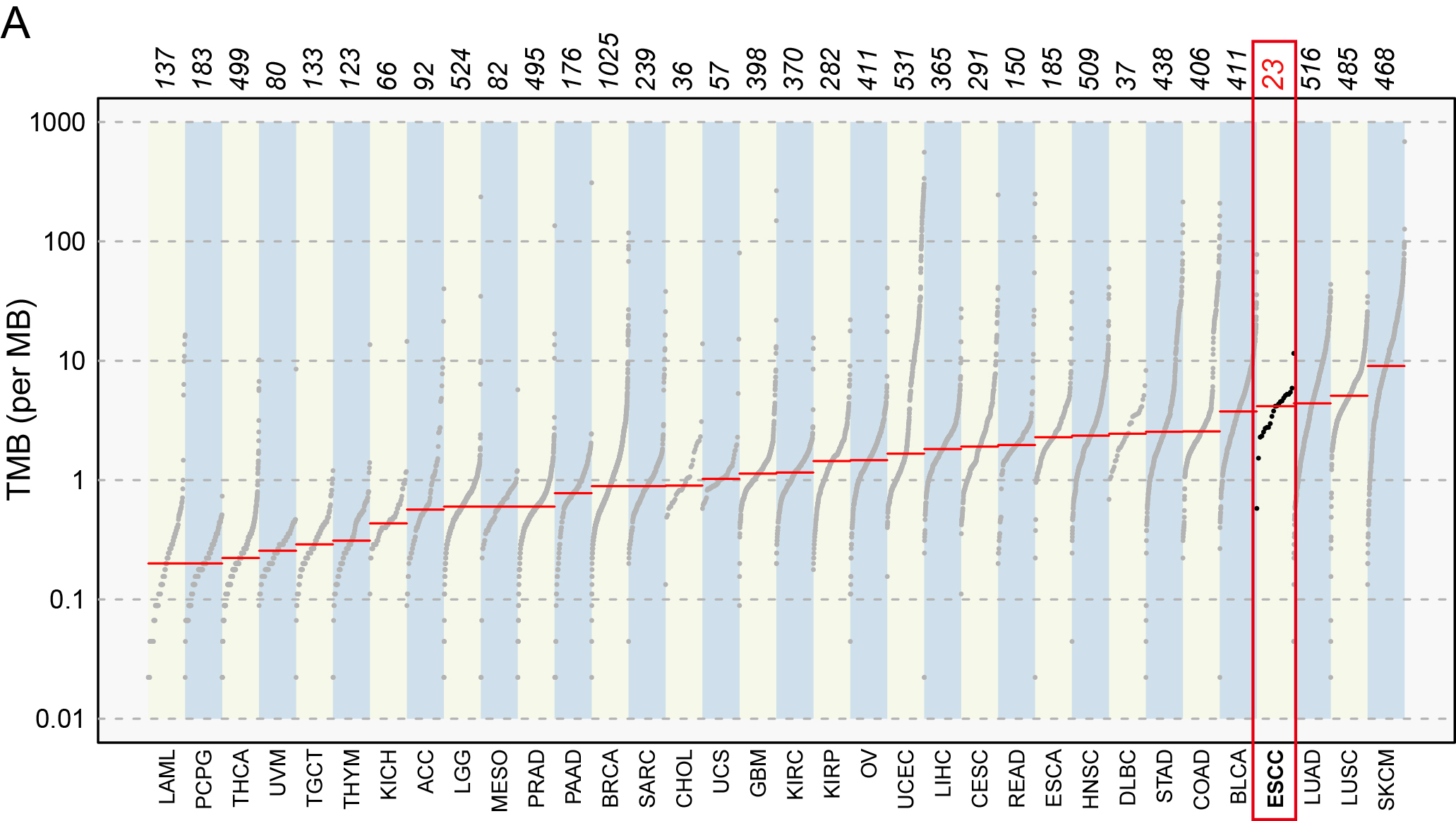

Supplement: Supplementary Figure 2 — The TMB of the 23 ESCC patients in this study and other cancers derived from TCGA. The numerical values above the figure indicate the total sample size for each cancer cohort. ESCC, esophageal squamous cell carcinoma; TMB, tumor mutation burden. [file Image2.jpeg]

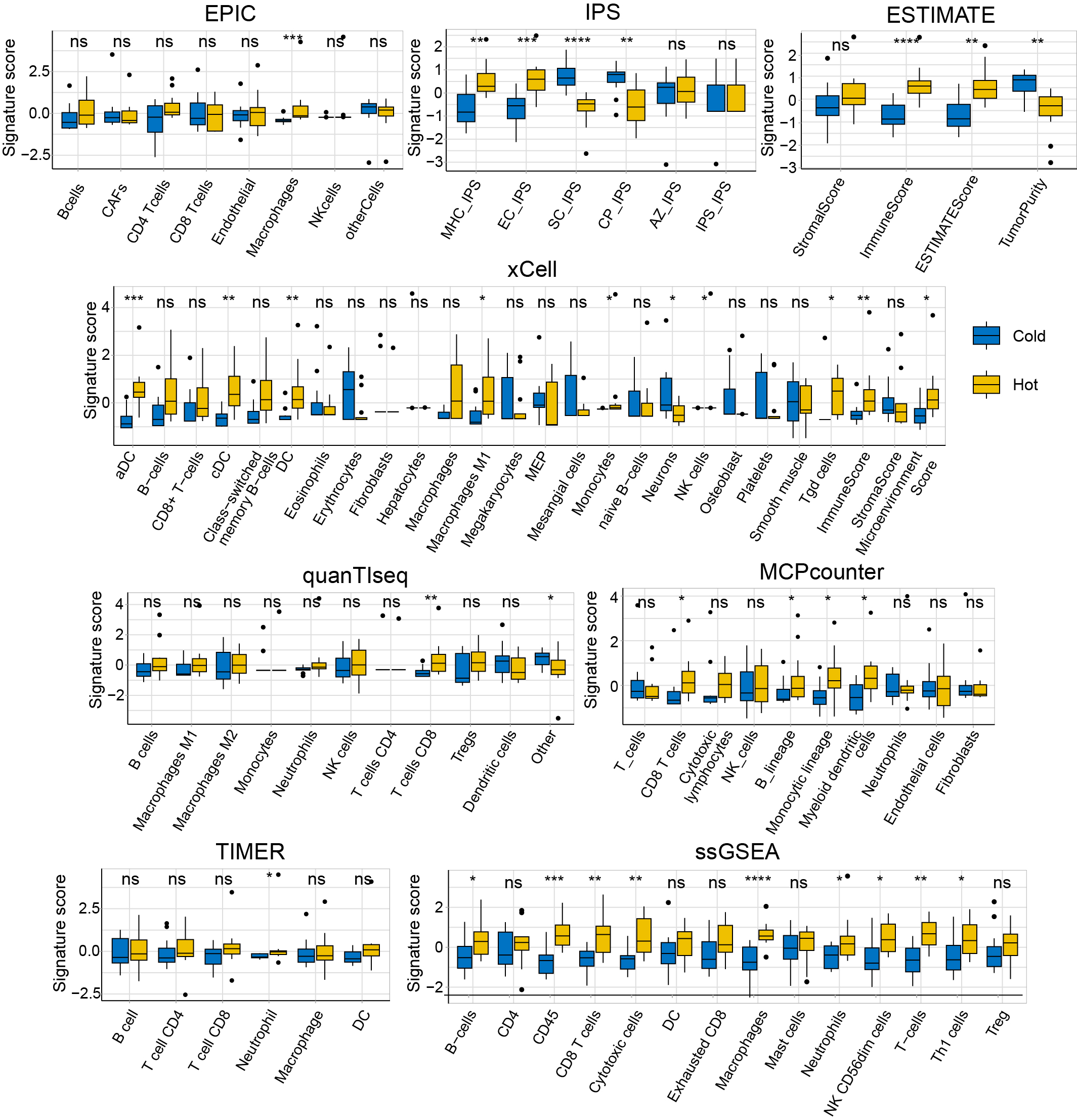

Supplement: Supplementary Figure 3 — Differential expression analysis of immune cell between PFS-H and PFS-L groups using multiple methodologies. ns, p≥0.05; **, p<0.01; ***, p<0.001. [file Image3.jpeg]

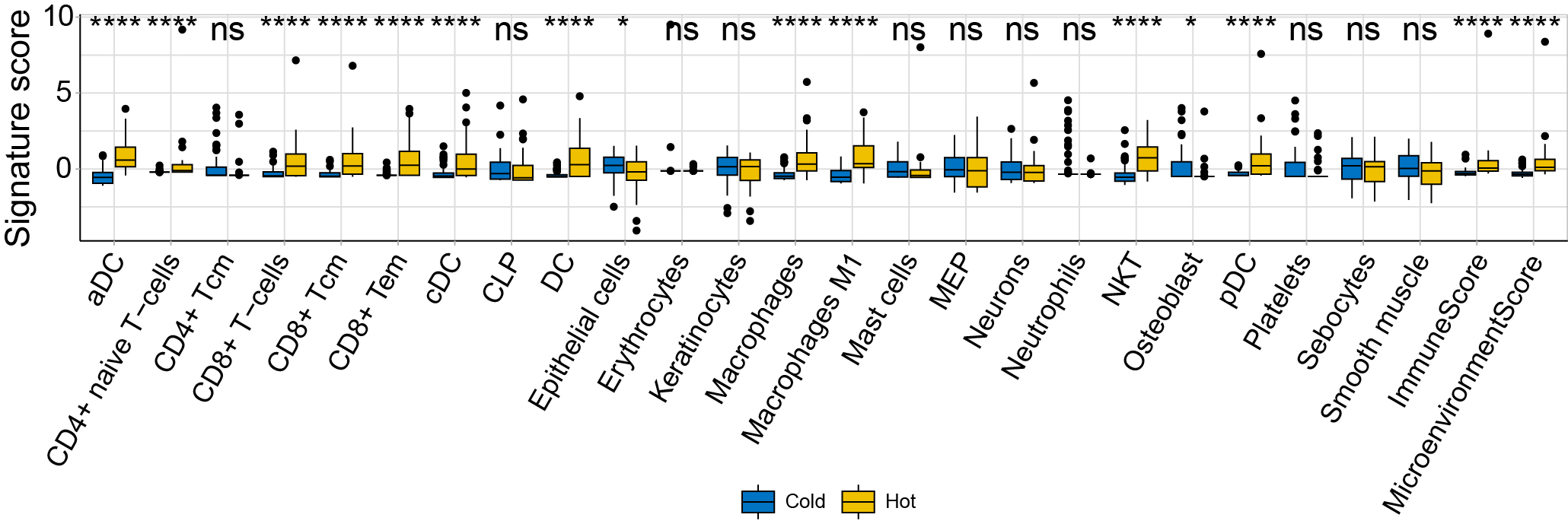

Supplement: Supplementary Figure 4 — The association of the immune infiltration derived from TCGA-ESCA database RNA sequencing. ns, p≥0.05; *, p<0.05; **, p<0.01; ***, p<0.001; ****, p < 0.0001. [file Image4.jpeg]

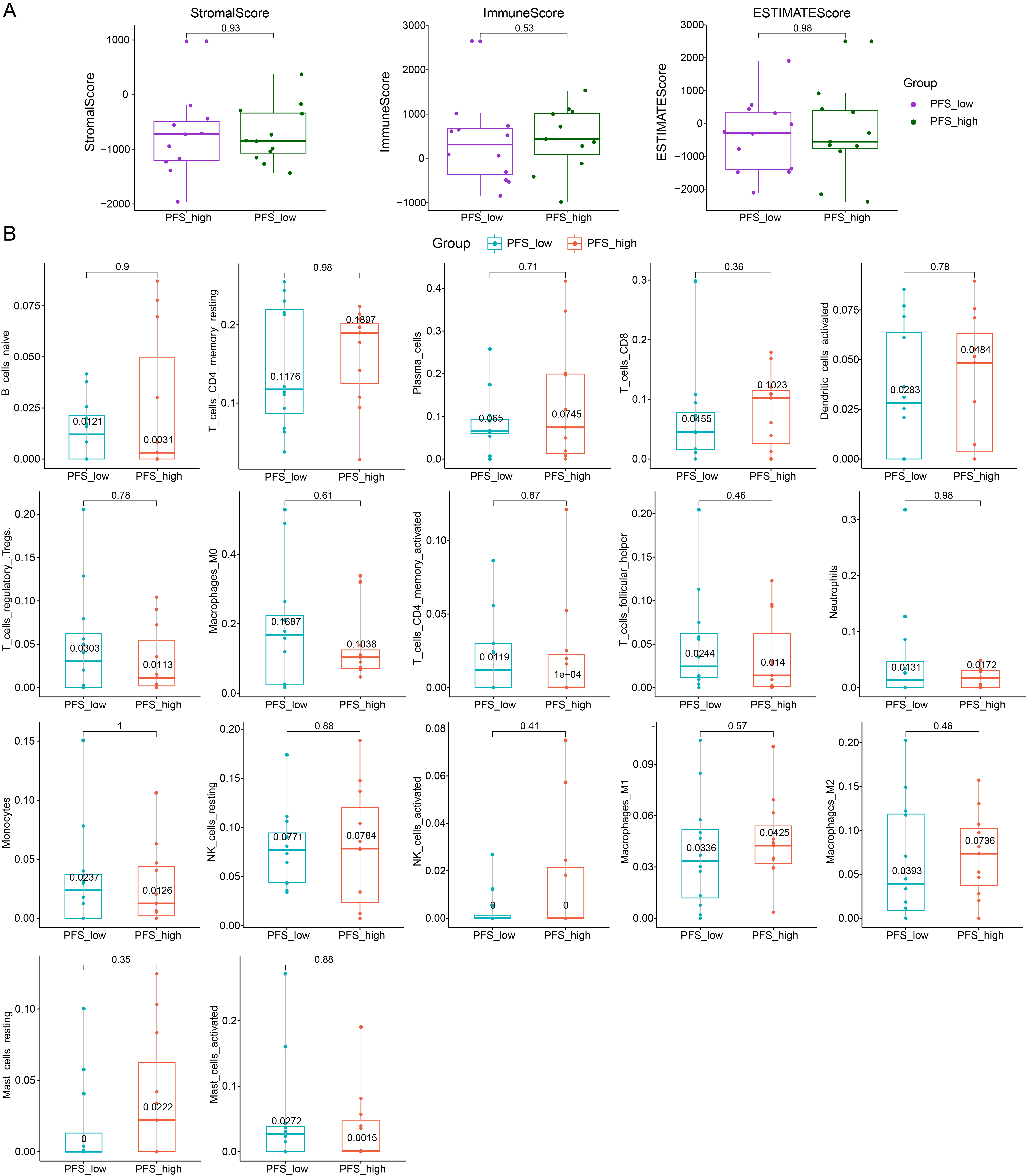

Supplement: Supplementary Figure 5 — The Immune-Score and the ratio of immune cells in PFS-high group and PFS-low group. (A) the ImmuneScore, StromalScore and ESTIMATEScore, in PFS-high group and PFS-low group; (B) The ratio of 22 immune cells between PFS-high group and PFS-low group. The numbers in the boxplots represent the median scores of each group. PFS, progression free survival. [file Image5.jpeg]

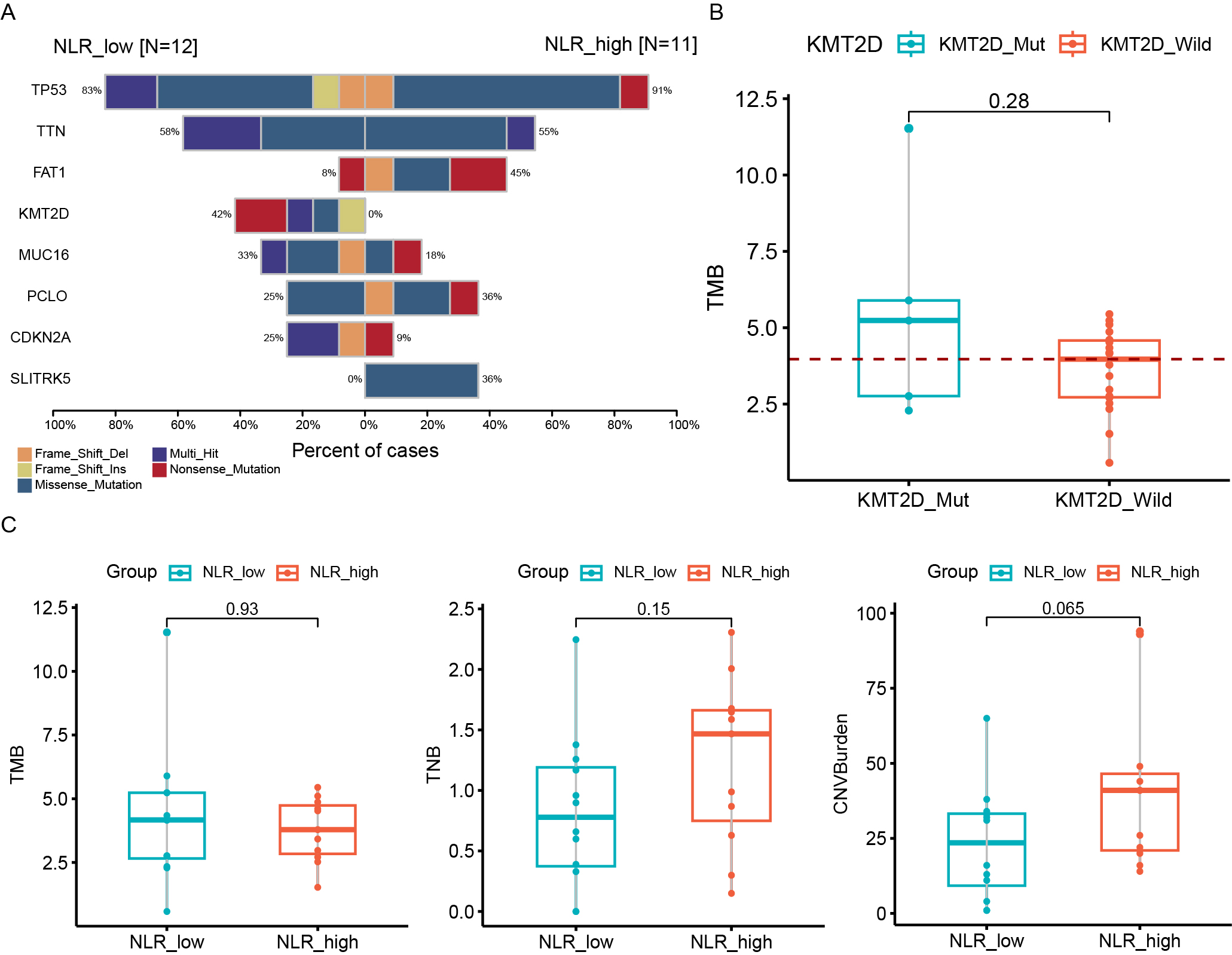

Supplement: Supplementary Figure 6 — The association of genomic characteristics between different NLRs (neutrophil-to-lymphocyte ratio). (A) The incidence of mutations in NLR-high group and NLR-low group; (B) The association between mutated gene and tumor mutational burden, KMT2D mutant (KMT2D-mut) samples tended to have higher TMB than KMT2D wild-type (KMT2D- wild) samples; (C) The difference in the median TMB, TNB and the chromosomal CNV burden between NLR-high group and NLR-low group. The numbers in the figure represent the p-values obtained from the Wilcoxon rank-sum test. (D) The Catalogue of Somatic Mutations in Cancer (COSMIC) mutational signatures composition in patients of NLR-low group (left panel) and NLR-high group (right panel). NLR, neutrophil-to-lymphocyte ratio; TMB, tumor mutation burden; TNB, tumor neoantigen burden; CNV, copy number variant. [file Image6.jpeg]

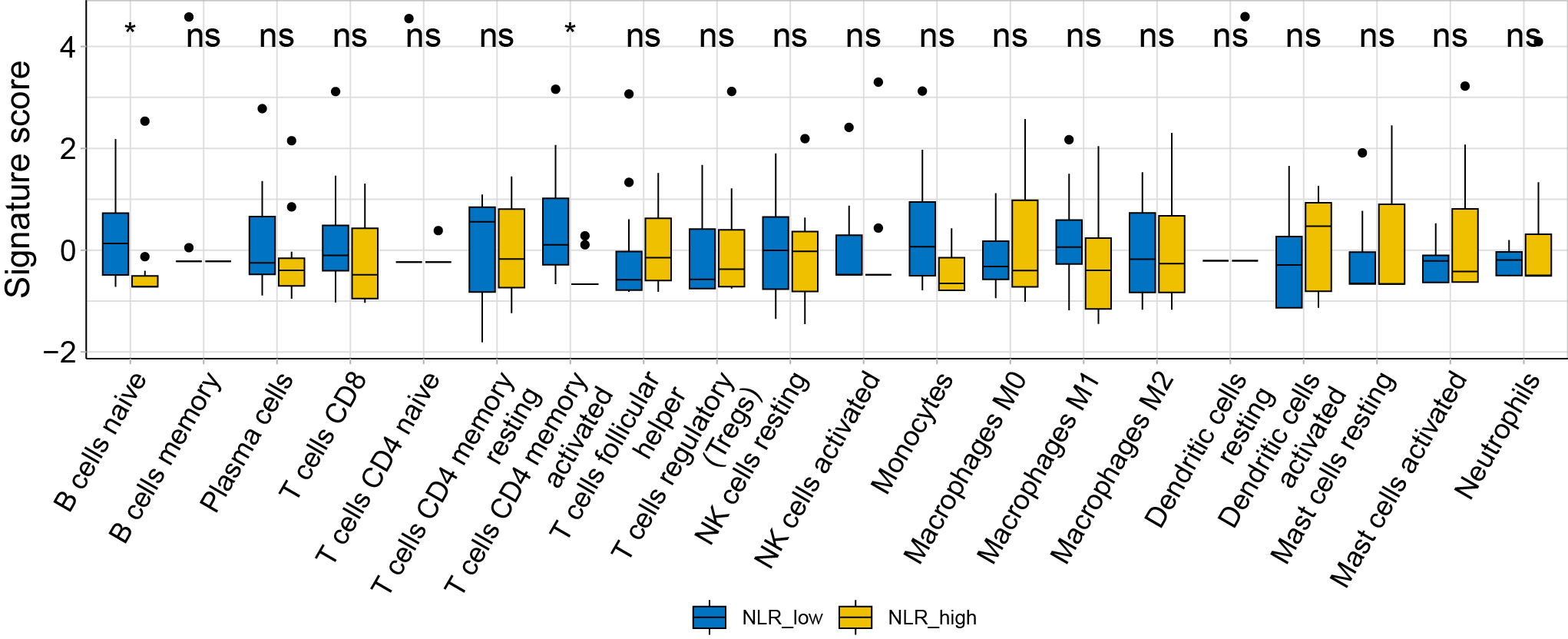

Supplement: Supplementary Figure 7 — The differences of the composition of immune cells between NLR-high group and NLR-low group. NLR, neutrophil-to-lymphocyte ratio; ns, p≥0.05; **, p<0.01; ***, p<0.001. [file Image7.jpeg]

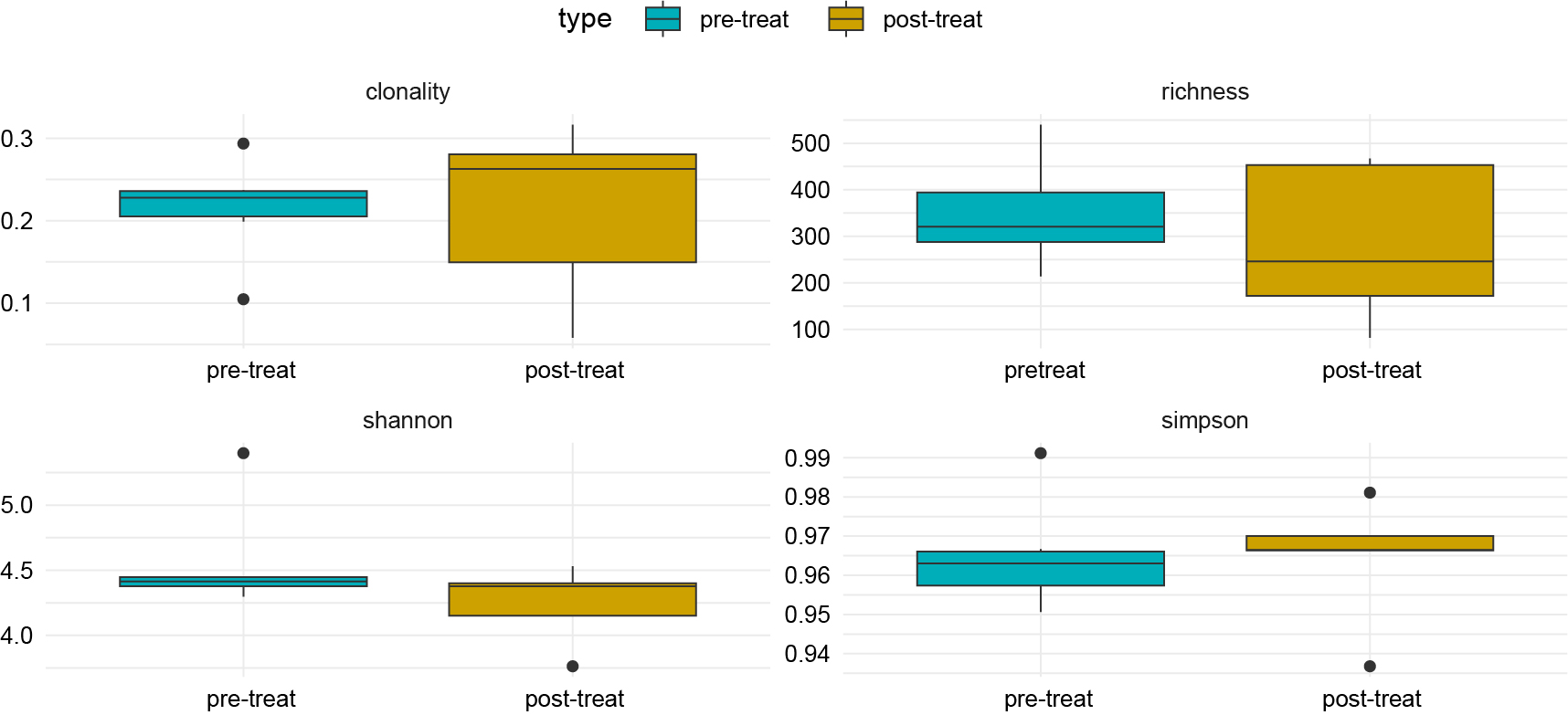

Supplement: Supplementary Figure 8 — TCRs dynamics verification by an external GSE120101 dataset of solid tumors. [file Image8.jpeg]

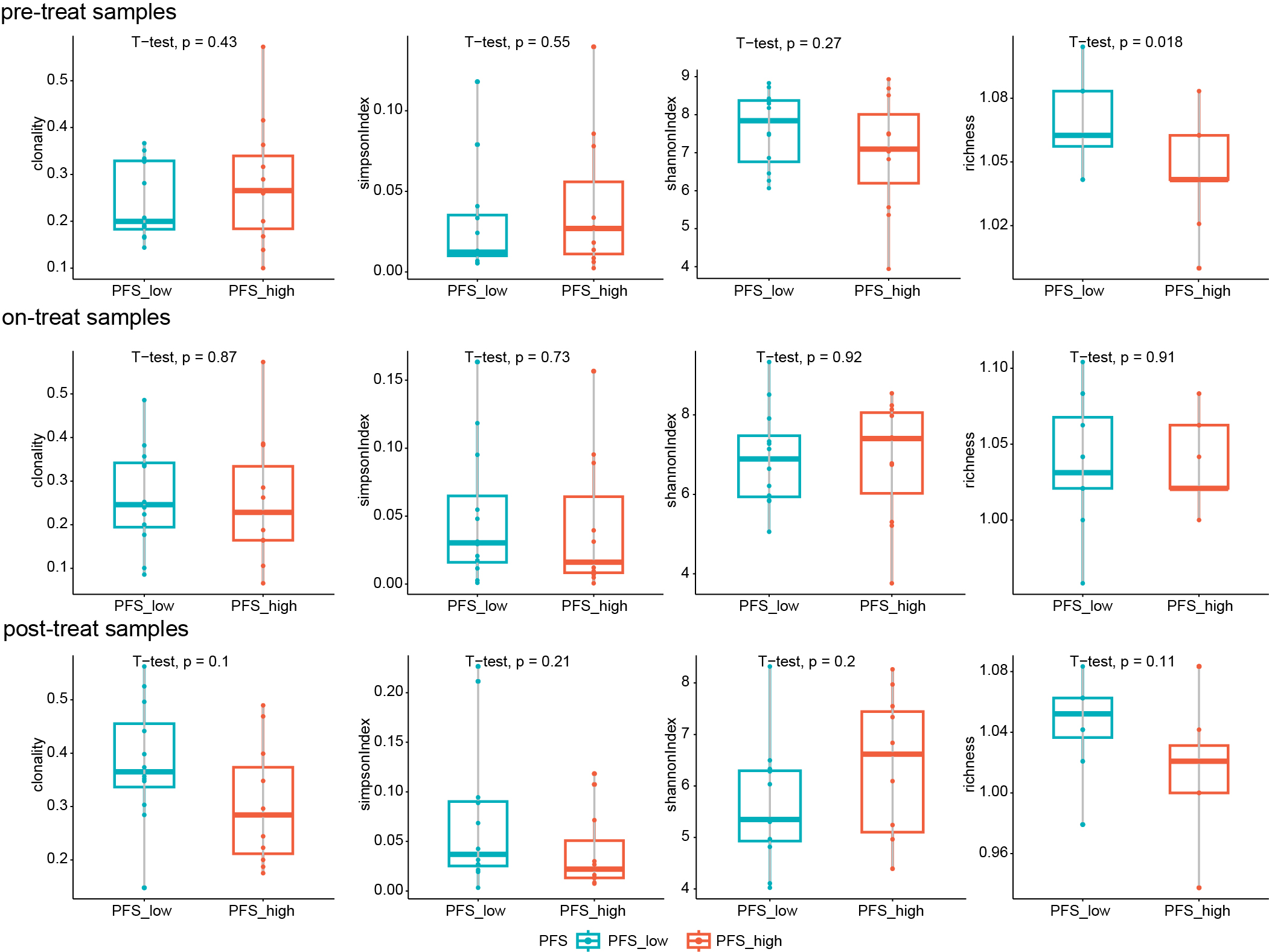

Supplement: Supplementary Figure 9 — The differences of the TCR clonality, simpson Index, shannon index and richness between NLR-high group and NLR-low group in the pre-treat, on-treat, or post-treat samples. TCR, T-cell receptors; NLR, neutrophil-to-lymphocyte ratio; pre-treat, pre-treatment; on-treat, on-treatment; post-treat, post-treatment. [file Image9.jpeg]

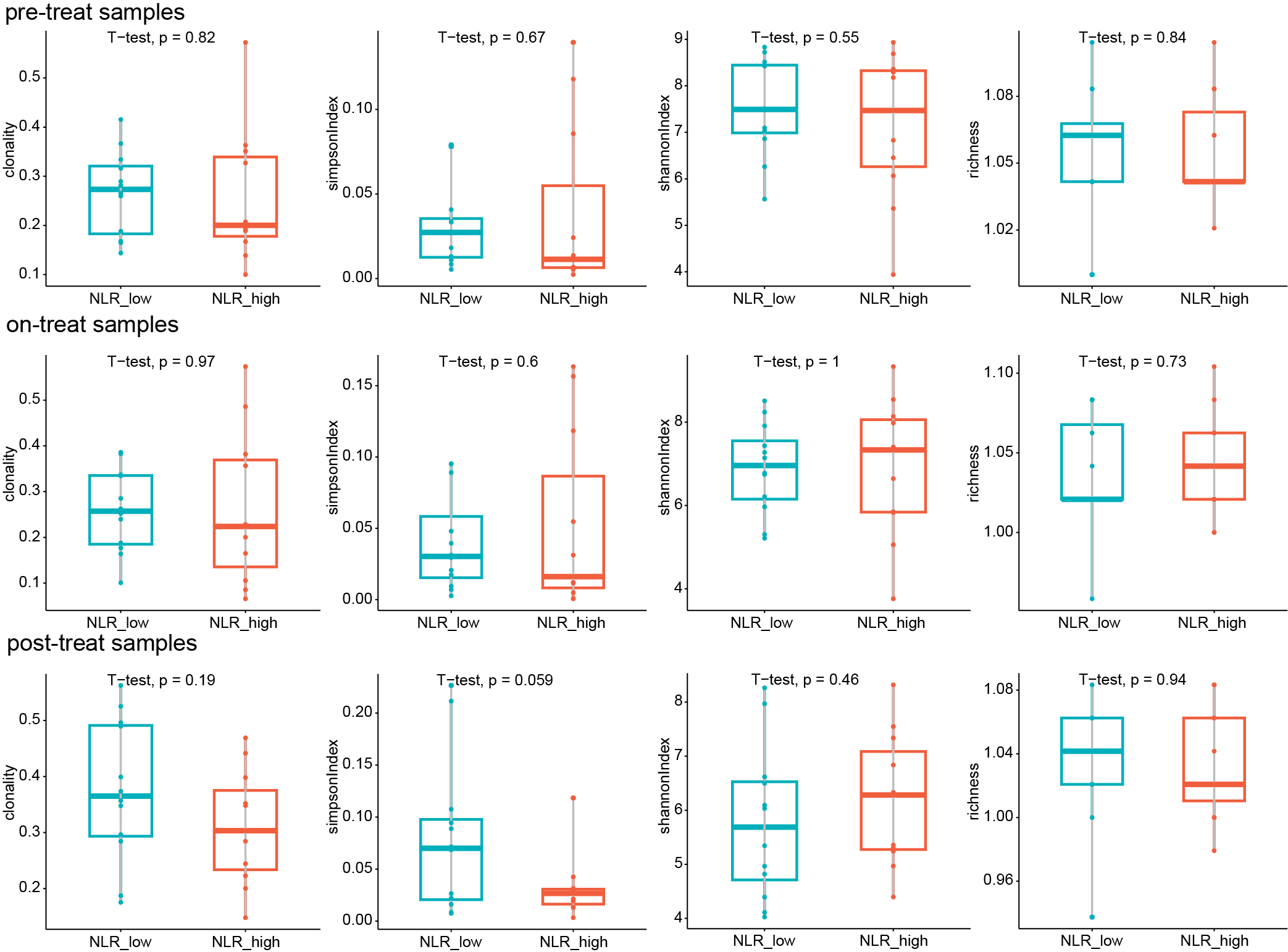

Supplement: Supplementary Figure 10 — The differences of the TCR clonality, simpson Index, shannon index and richness between PFS-high group and PFS-low group in the pre-treat, on-treat, or post-treat samples. TCR, T-cell receptors; PFS, progression free survival; pre-treat, pre-treatment; on-treat, on-treatment; post-treat, post-treatment. [file Image10.jpeg]

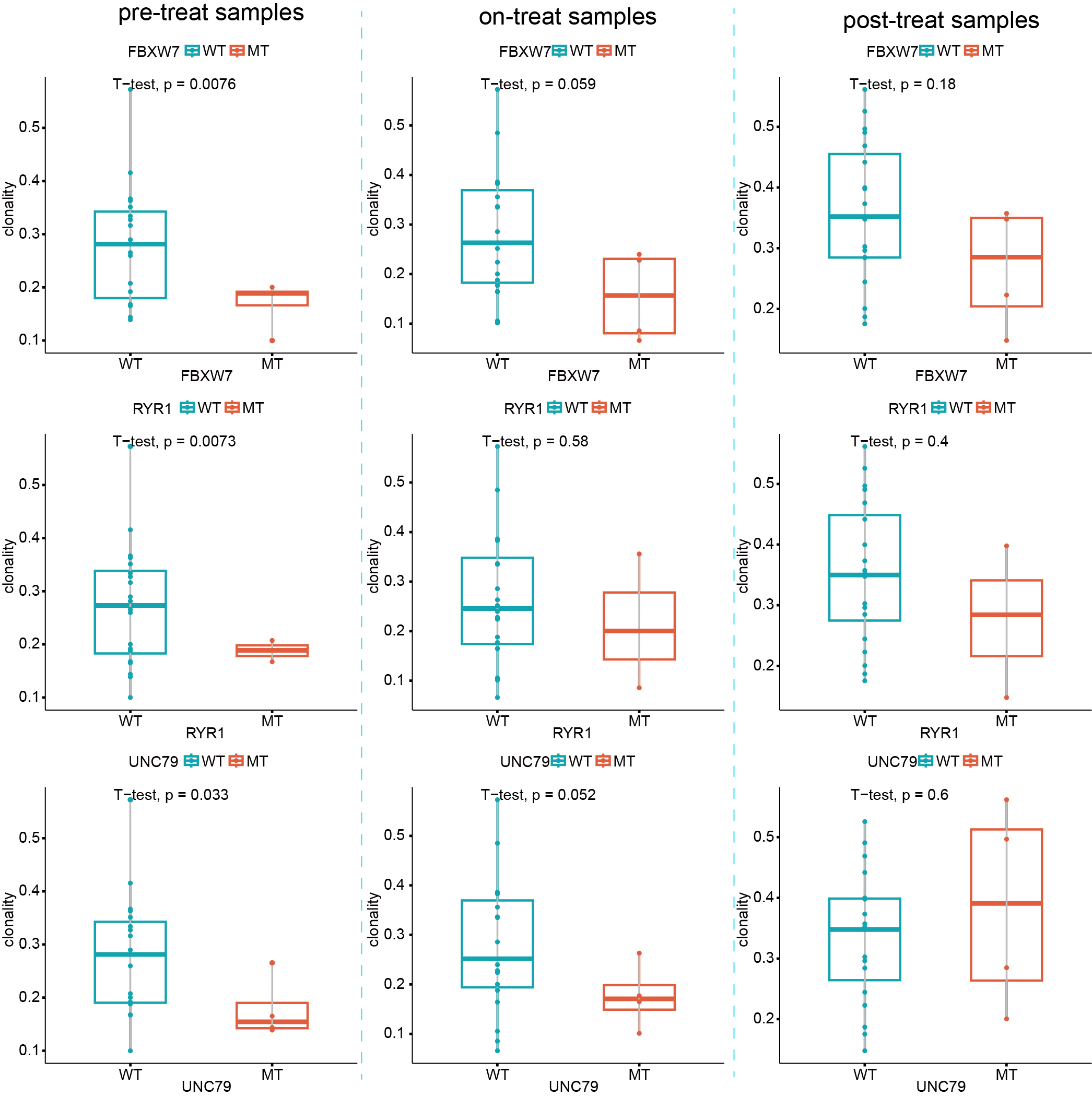

Supplement: Supplementary Figure 11 — The association between the Clonality and mutated genes. [file Image11.jpeg]
